# Melting, smelting, and recycling: A regional study around the Late Bronze Age mining site of Prigglitz-Gasteil, Lower Austria

**DOI:** 10.1371/journal.pone.0254096

**Published:** 2021-07-16

**Authors:** Marianne Mödlinger, Peter Trebsche, Benjamin Sabatini

**Affiliations:** 1 Dipartimento di Chimica e Chimica Industriale (DCCI), Università degli Studi di Genova, Genova, Italy; 2 Institut für Archäologien, Leopold-Franzens-Universität Innsbruck, Innsbruck, Austria; 3 Department of Mechanical Engineering, College of Science and Engineering, Ritsumeikan University Biwako-Kusatsu Campus (BKC), Kusatsu, Shiga, Japan; University of Vigo, SPAIN

## Abstract

This paper presents a study on copper production and distribution in Lower Austria’s southeastern region during the Late Bronze Age (c. 1350–800 BC), with the focal point being the chemistry and isotopic character of artifacts from a small copper mining site at Prigglitz-Gasteil on the Eastern Alps’ easternmost fringe. Ores, casting cakes, and select objects from the Late Bronze Age mining site at Prigglitz-Gasteil, Lower-Austria, and within 15 km of its surroundings, were chemically and isotopically analysed using XRF, NAA, and MC-ICPMS. The importance of Prigglitz-Gasteil as a local mining and metal processing center is evaluated based on the produced data, and the distribution and sourcing of copper-producing materials found at the site are discussed. Special attention is paid to the mixing of scrap and source materials early in the metal production process. The most salient discussions focus on the variability of the chemistry and Pb isotopic ratios of the studied objects, which seem to constitute a multitude of source materials, unlike the pure chalcopyrite-source copper produced from the Prigglitz-Gasteil mine itself. The analytical data suggests that copper alloys were mainly imported from materials originating in the Slovakian Ore Mountains, which were subsequently mixed/recycled with relatively pure locally produced copper. The purity of the copper from Prigglitz-Gasteil was fortuitous in identifying imported copper that contained measurable amounts of Pb and other chemically distinct characteristics. The *chaîne opératoire* of metal production at the site is mentioned; however, it is clear that additional information on the region’s geochemistry is required before any finite conclusions on the ore-to-metal production can be made.

## 1. Introduction

The production of metal and its distribution contributed to the social development of the European Bronze Age [1–4]; copper produced in the Eastern Alps was undoubtedly an important contributor, enabling an active and flourishing metals trade in the region [5, 6]. For a general overview of European copper mining, see [7]. The organization of copper production and the mechanisms of distribution have been intensively studied in large-scale metal producing regions like the Mitterberg, where peak copper production was reached in the Middle Bronze Age (MBA, ca 1650–1350 BC) [8–12]; however, very little is known about the later emergence of small copper-producing sites in the Late Bronze Age (LBA, ca 1350–800 BC), the decline of activity at the Mitterberg mine, and the returned use of fahlores in central Europe. Indeed, despite the availability of large datasets of copper-based alloy analyses [13], little progress has been made in understanding the complete picture of trade and technological progression in Europe. Only when interdisciplinary evaluations of data that consider the chemistry, Pb isotopic ratios, and chronology and contexts of samples throughout the *chaîne opératoire* at individual sites will headway be made to decipher the larger picture of metal production in Europe. The reliability of such studies depends largely upon the characterisation of the investigated source materials, too (cf. [14]), and is the approach taken by this work in studying Prigglitz-Gasteil.

**Fig 1:**
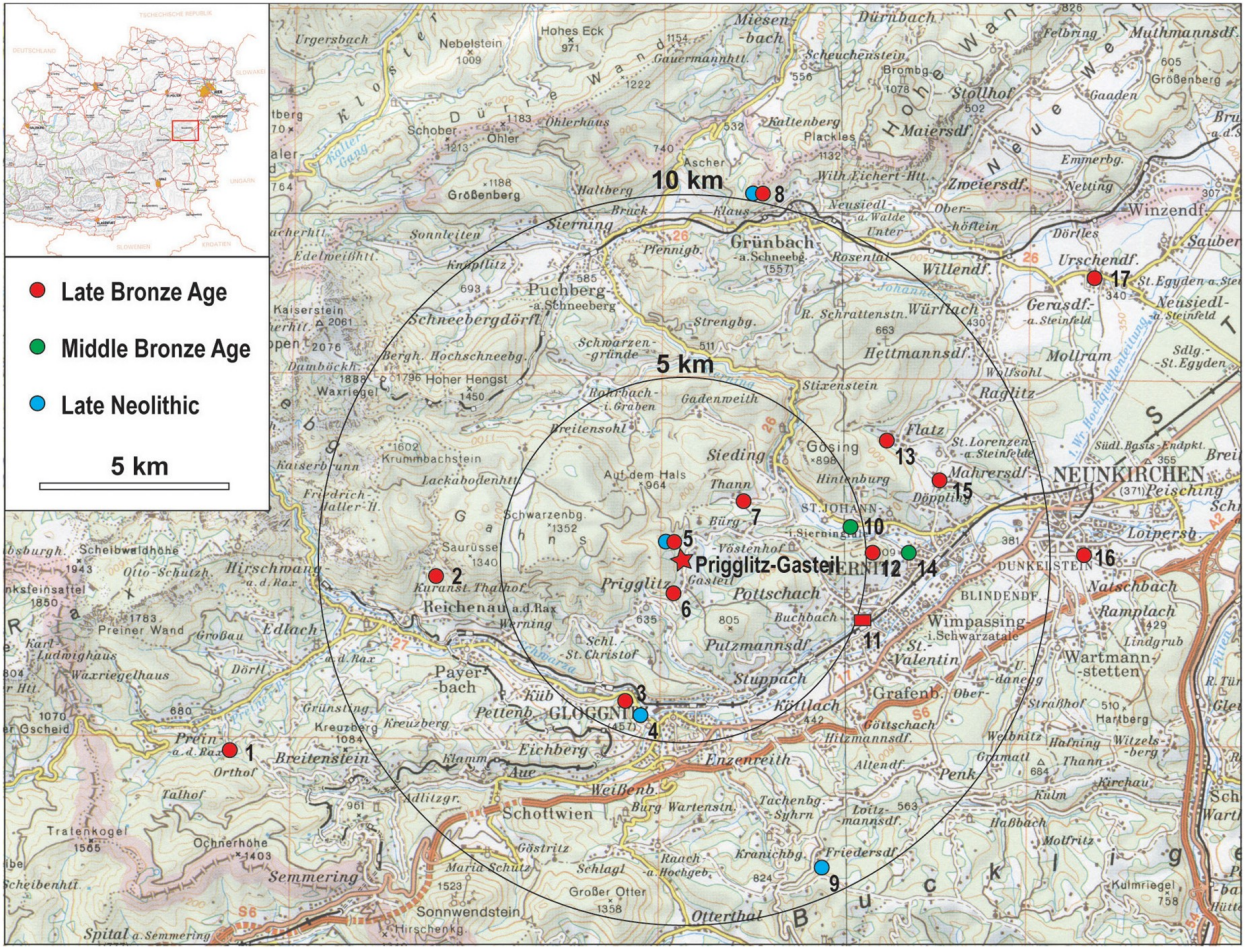
Location of Prigglitz-Gasteil and the surrounding sample findspots. Cartography from the Bundesamt für Eich- und Vermessungswesen (BEV) with kind permission from BEV, original copyright [2021]. 1 Prein an der Rax; 2 Reichenau-Kammerwandgrotte; 3 Heufeld-Heukogel; 4 Gloggnitz (Semmeringtunnelportal); 5 Prigglitz-Gasteil/Bürg (Klausgraben); 6 Prigglitz-Gasteil (Kapelle); 7 Sieding (Murrer); 8 Grünbach am Schneeberg-Gelände; 9 Kranichberg-Karlhöhe; 10 Sieding; 11 Pottschach; 12 Ternitz-Gfieder; 13 Flatz-Gösing; 14 Ternitz-St. Johann; 15 Mahrersdorf; 16 Neunkirchen/Natschbach-Klosteräcker; 17 Urschendorf.

This paper presents a thorough archaeometallurgical investigation of a small LBA copper mining site in southeastern Lower Austria at Prigglitz-Gasteil on the easternmost fringe of the Eastern Alps. In addition to 11 ore samples and 34 excavated copper alloy artefacts from Prigglitz-Gasteil (Table 1), we have analysed 4 ores from Prein an der Rax and 76 artefacts from contemporary contexts within an ~ 15 km radius around the site (Fig 1). With these newly acquired data, we can begin to reconstruct the circulation of copper ore, raw metal, metal objects, and technological knowledge not only at the intra-site level but in the surrounding region of southeastern Lower Austria. We consider each step in the metal-making process, from mining the ore to the distribution of finished objects (for a recent overview of the state of research and current debates, see [14, 15]. We also consider the possible influx of metal from outside Prigglitz, including other copper ores, imported tin, and recycled copper-based scrap. With these considerations in mind, the aims of this paper are: 1) to characterize the locally produced copper at the Prigglitz-Gasteil site; 2) to identify products of the Prigglitz-Gasteil mine in a regional context; 3) to identify copper-alloy objects of different origin at Prigglitz-Gasteil; and, 4) to investigate the local *chaîne opératoire* of copper production.

**Table 1:** Objects analysed in this paper.

No.	ID / Inv.no.	Collection	Find spot	Object	Chronology	Reference	Figure
1	UF-22692.631	LSNÖ	Prigglitz-Gasteil, site "Cu I"	casting cake	Urnfield Culture (Ha B)	unpublished	Fig 3/1
2	UF-10.432 (S073)	LSNÖ	Prigglitz-Gasteil, site "Cu I"	casting cake	Urnfield Culture (Ha B)	unpublished	Fig 3/2
3	UF-10.862 (S074)	LSNÖ	Prigglitz-Gasteil, site "Cu I"	casting cake	Urnfield Culture (Ha B)	unpublished	Fig 3/3
4	UF-10.921 (S075)	LSNÖ	Prigglitz-Gasteil, site "Cu I"	casting cake	Urnfield Culture (Ha B)	unpublished	Fig 3/4
5	UF-22692.675	LSNÖ	Prigglitz-Gasteil, site "Cu I"	casting cake	Urnfield Culture (Ha B)	unpublished	Fig 3/5
6	not inv. (GAST05)	LSNÖ	Prigglitz-Gasteil, site "Cu I"	casting cake	Urnfield Culture (stray find)	unpublished	-
7	not inv. (GAST06)	LSNÖ	Prigglitz-Gasteil, site "Cu I"	casting cake	Urnfield Culture (stray find)	unpublished	-
8	not inv. (S068)	LSNÖ	Prigglitz-Gasteil, site "Cu I"	casting cone	Urnfield Culture (stray find)	unpublished	Fig 3/8
9	UF-22692.187	LSNÖ	Prigglitz-Gasteil, site "Cu I"	casting sprue	Urnfield Culture (Ha B)	[16], Fig 14/2	Fig 3/9
10	UF-22692.1099	LSNÖ	Prigglitz-Gasteil, site "Cu I"	casting drop	Urnfield Culture (Ha B)	unpublished	-
11	UF-22692.2183	LSNÖ	Prigglitz-Gasteil, site "Cu I"	casting drop	Urnfield Culture (Ha B)	unpublished	-
12	UF-22692.10	LSNÖ	Prigglitz-Gasteil, site "Cu I"	arrowhead (socketed)	Urnfield Culture (Ha B)	[16], Fig 19/5	Fig 3/12
13	UF-22692.792	LSNÖ	Prigglitz-Gasteil, site "Cu I"	arrowhead (socketed)	Urnfield Culture (Ha B)	[16], Fig 19/4	Fig 3/13
14	UF-22692.30	LSNÖ	Prigglitz-Gasteil, site "Cu I"	ring	Urnfield Culture (Ha B)	[16], Fig 19/3	Fig 3/14
15	UF-22692.789	LSNÖ	Prigglitz-Gasteil, site "Cu I"	ring	Urnfield Culture (Ha B)	[16], Fig 19/2	Fig 3/15
16	UF-10.964	LSNÖ	Prigglitz-Gasteil, site "Cu I"	knife	Urnfield Culture (Ha B)	[17], 76 no. 303 pl. 29/303; [18], Fig 2/5	Fig 3/16
17	UF-22692.2188	LSNÖ	Prigglitz-Gasteil, site "Cu I"	knife (type Griffangel)	Urnfield Culture (Ha B)	[18], Fig 2/6	Fig 3/17
18	UF-22692.913	LSNÖ	Prigglitz-Gasteil, site "Cu I"	pin (type Kugelkopf)	Urnfield Culture (Ha B)	[16], Fig 19/1	Fig 3/18
19	UF-22692.1853	LSNÖ	Prigglitz-Gasteil, site "Cu I"	pin (type Nagelkopf)	Urnfield Culture (Ha B)	unpublished	Fig 3/19
20	UF-22692.881	LSNÖ	Prigglitz-Gasteil, site "Cu I"	awl	Urnfield Culture (Ha B)	[16], Fig 19/11	Fig 3/20
21	UF-22692.1140A	LSNÖ	Prigglitz-Gasteil, site "Cu I"	awl	Urnfield Culture (Ha B)	[16], Fig 19/10	Fig 3/21
22	UF-22692.1272	LSNÖ	Prigglitz-Gasteil, site "Cu I"	awl	Urnfield Culture (Ha B)	unpublished	Fig 3/22
23	UF-22692.1672	LSNÖ	Prigglitz-Gasteil, site "Cu I"	awl	Urnfield Culture (Ha B)	unpublished	Fig 3/23
24	UF-22692.413	LSNÖ	Prigglitz-Gasteil, site "Cu I"	awl	Urnfield Culture (Ha B)	unpublished	Fig 3/24
25	UF-22692.666	LSNÖ	Prigglitz-Gasteil, site "Cu I"	awl	Urnfield Culture (Ha B)	unpublished	Fig 3/25
26	UF-22692.1673	LSNÖ	Prigglitz-Gasteil, site "Cu I"	clip (belt)	Urnfield Culture (Ha B)	unpublished	Fig 3/26
27	UF-22692.1652	LSNÖ	Prigglitz-Gasteil, site "Cu I"	clip (belt)	Urnfield Culture (Ha B)	unpublished	Fig 3/27
28	UF-22692.912	LSNÖ	Prigglitz-Gasteil, site "Cu I"	bar (fragment)	Urnfield Culture (Ha B)	[16], Fig 19/6	Fig 3/28
29	UF-22692.1854	LSNÖ	Prigglitz-Gasteil, site "Cu I"	bar (fragment)	Urnfield Culture (Ha B)	unpublished	Fig 3/29
30	UF-22692.303	LSNÖ	Prigglitz-Gasteil, site "Cu I"	wire (fragment)	Urnfield Culture (Ha B)	unpublished	Fig 3/30
31	UF-22692.1780	LSNÖ	Prigglitz-Gasteil, site "Cu I"	bracelet (fragment)	Urnfield Culture (Ha B)	unpublished	Fig 3/31
32	UF-22692.1110	LSNÖ	Prigglitz-Gasteil, site "Cu I"	tubelet (fragment)	Urnfield Culture (Ha B)	unpublished	Fig 3/32
33	UF-22692.2177	LSNÖ	Prigglitz-Gasteil, site "Cu I"	tip (fragment)	Urnfield Culture (Ha B)	unpublished	Fig 3/33
34	UF-22692.2335	LSNÖ	Prigglitz-Gasteil, site "Cu I"	bar (fragment)	Urnfield Culture (Ha B)	unpublished	Fig 3/34
35	S005	Private	Prigglitz-Gasteil, Kapelle	spear head	Bronze Age (stray find)	unpublished	-
36	S021	Private	Prigglitz-Gasteil, Kapelle	pin	Bronze Age (stray find)	unpublished	-
37	S001	Private	Prigglitz-Gasteil, Klausgraben	axe (socketed)	Urnfield Culture (Ha B)	[18], 47 Fig 2/3	Fig 4/37
38	S044	Private	Bürg near Prigglitz-Gasteil, Klausgraben	axe (flat)	Late Neolithic	[19], 13 Fig 13	-
39	UF-22692.730	LSNÖ	Prigglitz-Gasteil, site "Cu I"	copper ore		unpublished	-
40	UF-22692.892	LSNÖ	Prigglitz-Gasteil, site "Cu I"	copper ore		unpublished	-
41	UF-22692.1142	LSNÖ	Prigglitz-Gasteil, site "Cu I"	copper ore		unpublished	-
42	UF-22692.2514	LSNÖ	Prigglitz-Gasteil, site "Cu I"	copper ore		unpublished	-
43	UF-22692.2553	LSNÖ	Prigglitz-Gasteil, site "Cu I"	copper ore		unpublished	-
44	UF-22692.2558	LSNÖ	Prigglitz-Gasteil, site "Cu I"	copper ore		unpublished	-
45	UF-22692.2666	LSNÖ	Prigglitz-Gasteil, site "Cu I"	copper ore		unpublished	-
46	UF-22692.2678	LSNÖ	Prigglitz-Gasteil, site "Cu I"	copper ore		unpublished	-
47	UF-22692.2682	LSNÖ	Prigglitz-Gasteil, site "Cu I"	copper ore		unpublished	-
48	UF-10796.2	LSNÖ	Prigglitz-Gasteil, site "Cu I"	copper ore		unpublished	-
49	UF-22692.1150	LSNÖ	Prigglitz-Gasteil, site "Cu I"	copper ore		unpublished	-
50	S025	Private	Flatz-Gösing	spear head	Bronze Age	unpublished	-
51	S002	Private	Gloggnitz-Semmeringtunnelportal	axe (flat)	Late Neolithic	[20], 269 Fig 51	-
52	S014	Private	Grünbach-Gelände	copper fragment	unknown (stray find)	unpublished	-
53	S015	Private	Grünbach-Gelände	copper fragment	unknown (stray find)	unpublished	-
54	S016	Private	Grünbach-Gelände	copper fragment	unknown (stray find)	unpublished	-
55	S049	Private	Grünbach-Gelände	axe (end-winged)	Urnfield Culture (Ha B)	[21], Pl. 34/3	Fig 4/55
56	S050	Private	Grünbach-Gelände	chisel (socketed)	Urnfield Culture	[21], Pl. 34/5	Fig 4/56
57	S051	Private	Grünbach-Gelände	axe (socketed)	Urnfield Culture	[21], Pl. 34/7	Fig 4/57
58	S070	Private	Grünbach	axe (hammer-shaped)	Late Neolithic	unpublished	-
59	UF-19453	LSNÖ	Grünbach-Gelände	bracelet	Urnfield Culture	[22], 563 Fig 6	-
60	UF-19451	LSNÖ	Grünbach-Gelände	casting cake	Urnfield Culture (Ha B)	[22], 567 Fig 8	Fig 4/60
61	UF-19452	LSNÖ	Grünbach-Gelände	axe (end-winged)	Urnfield Culture (Ha B)	[22], 561 Fig 5	Fig 4/61
62	H001	Private	Heufeld	casting cake	Bronze Age	[21], 206 Taf. 94	-
63	H003	Private	Heufeld	casting cake	Bronze Age	[21], 206 Taf. 94	-
64	H004	Private	Heufeld	casting cake	Bronze Age	[21], 206 Taf. 94	-
65	H005	Private	Heufeld	casting cake	Bronze Age	[21], 206 Taf. 94	-
66	S071	Private	Kranichberg-Karlhöhe	axe (fragment)	Late Neolithic	unpublished	-
67	3720 (S067)	SMNK	Neunkirchen-Klosteräcker	casting cake	Urnfield Culture	[21], 206	Fig 4/67
68	9024 (S060)	SMNK	Neunkirchen-Klosteräcker	casting cake	Urnfield Culture	[21], 206	Fig 4/68
69	9027 (S063)	SMNK	Neunkirchen-Klosteräcker	casting cake	Urnfield Culture	[21], 206	-
70	9029 (S065)	SMNK	Neunkirchen-Klosteräcker	casting cake	Urnfield Culture	[21], 206	-
71	72.484	NHM-PA	Pottschach	knife	Urnfield Culture (Ha B)	[27], 68 no. 274 pl. 26/274	Fig 4/71
72	72.485	NHM-PA	Pottschach	knife	Urnfield Culture (Ha B)	[27], 52 no. 179 pl. 17/179	Fig 4/72
73	72.487 A	NHM-PA	Pottschach	pin (type Vasenkopf)	Urnfield Culture (Ha B)	[23], 198 no. 1609 pl. 59/1609	-
74	72.487 B	NHM-PA	Pottschach	pin (type Vasenkopf)	Urnfield Culture (Ha B)	[23], 198 no. 1610 pl. 59/1610	-
75	72.488	NHM-PA	Pottschach	pin (type Vasenkopf)	Urnfield Culture (Ha B)	[23], 203 no. 1706 pl. 62/1706	-
76	72.489	NHM-PA	Pottschach	bracelet	Urnfield Culture (Ha B)	[24], Fig 2	-
77	72.491 A	NHM-PA	Pottschach	bracelet	Urnfield Culture (Ha B)	[24], Fig 2	-
78	72.491 B	NHM-PA	Pottschach	bracelet	Urnfield Culture (Ha B)	[24], Fig 2	-
79	72.491 C	NHM-PA	Pottschach	bracelet	Urnfield Culture (Ha B)	[24], Fig 2	-
80	72.492 A	NHM-PA	Pottschach	bracelet	Urnfield Culture (Ha B)	[24], Fig 2	-
81	72.492 B	NHM-PA	Pottschach	bracelet	Urnfield Culture (Ha B)	[24], Fig 2	-
82	UF-9751 (S078)	LSNÖ	Prein, site III	pin (type Rippenkopf)	Urnfield Culture (Ha B)	[23], 219 no. 1823 pl. 66/1823; [18], Fig 2/1	-
83	UF-9958	LSNÖ	Prein, site V	awl	Urnfield Culture (Ha B)	[25], 52	-
84	UF-9723.1	LSNÖ	Prein, site II	copper ore		[26]	-
85	UF-9723.2	LSNÖ	Prein, site II	copper ore		[26]	-
86	UF-9722a.1	LSNÖ	Prein, site II	copper ore		[26]	-
87	UF-9970	LSNÖ	Prein, site VII	copper ore		[25], 53	-
88	not inv. (Fn. 24, S004, S041)	LSNÖ	Reichenau-Kammerwandgrotte	chisel (fragment)	Bronze Age	[27], Fig 249	Fig 4/88
89	not inv. (S010)	LSNÖ	Reichenau-Kammerwandgrotte	casting residue	Bronze Age	unpublished	-
90	not inv. (S011)	LSNÖ	Reichenau-Kammerwandgrotte	casting residue	Bronze Age	unpublished	-
91	not inv. (S013)	LSNÖ	Reichenau-Kammerwandgrotte	wire	Bronze Age	[27], Fig 248	-
92	not inv. (S069)	Private	Reichenau-Kammerwandgrotte	casting cake	Bronze Age	unpublished	-
93	UF-4553 (S033)	LSNÖ	Sieding-Hügel 1	belt	Middle Bronze Age	[28], 100 no. 397 pl. 36-37/397	-
94	UF-4554 (S036)	LSNÖ	Sieding-Hügel 1	pin (type Lochhals)	Middle Bronze Age	[23], 31 no. 81 pl. 5/81	-
95	UF-4555 (S037)	LSNÖ	Sieding-Hügel 1	pin (type Lochhals)	Middle Bronze Age	[23], 31 no. 82 pl. 5/82	-
96	UF-4957	LSNÖ	Sieding-Hügel 1	pin (type Lochhals)	Middle Bronze Age	[23], 30 no. 77 pl. 5/77	-
97	UF-4958	LSNÖ	Sieding-Hügel 1	pin (type Lochhals)	Middle Bronze Age	[23], 23 no. 39 pl. 3/39	-
98	UF-4959 (S012)	LSNÖ	Sieding-Hügel 1	ring	Middle Bronze Age	[29], 398 pl. 31/3	-
99	UF-5098	LSNÖ	Sieding-Murrer	axe (socketed)	Urnfield Culture (Ha B)	[30], 204 no. 1175 Taf. 84/1175	Fig 4/99
100	UF-6117 (S023)	LSNÖ	Sieding-Steinparzkreuz	casting cake	Bronze Age	[29], 399	-
101	not inv. (S003)	Private	Ternitz-Gfieder	axe (socketed)	Urnfield Culture (Ha B)	[31], 599 Abb. 474	Fig 4/101
102	UF-9019 (S076)	LSNÖ	Ternitz-St. Johann	pin	Middle Bronze Age	[23], 23 no. 40 pl. 3/40	-
103	not inv. (Fn. 1, S008)	BDA	Urschendorf	pin (type Vasenkopf)	Urnfield Culture (Ha B)	unpublished	-
104	not inv. (Fn. 1, S009)	BDA	Urschendorf	tip (fragment)	Urnfield Culture (Ha B)	unpublished	-
105	not inv. (Fn. 32, S007)	BDA	Urschendorf	pin (type Zylinderkopf)	Urnfield Culture (Ha B)	unpublished	-
106	not inv. (Fn. 7, S006)	BDA	Urschendorf	pin (type Rollenkopf)	Early Iron Age (Ha C)	[32], 326 Fig 42.	-

Collections: Landessammlungen Niederösterreich (LSNÖ); Stadtmuseum Neunkirchen (SMNK); Bundesdenkmalamt Österreich (BDA); Natural History Museum Vienna, Department of Prehistory (NHM-PA).

The results of this study are intended to contribute to an understanding of two significant trends in LBA copper production at Prigglitz-Gasteil and the surrounding region: first, the emergence of many small and geographically dispersed copper producers in the Alps; and second, the European trend towards the exploitation of fahlores and the production of highly impure copper. Despite containing relatively less pure copper, many small fahlore mines superseded larger underground chalcopyrite ones, like those at Mitterberg that were heavily exploited during the MBA. Thomas Stöllner labeled this period of geographical expansion and dispersion the "second zenith," which was followed by the decline of Alpine copper production at the beginning of the Early Iron Age [5, 33]. Of note, and in contrast with this general trend, the Prigglitz mine predominantly contains chalcopyrite, yet it was exploited contemporaneously with several major and minor LBA fahlore sites in the Slovakian Ore Mountains, the Tyrolean Lower Inn Valley, and in the Rax-, Hohe Wand-, and Kőszeg-Güns-mountain regions [34–36].

## 2. Materials and methods

### 2.1 The Late Bronze Age mining site at Prigglitz

**Fig 2:**
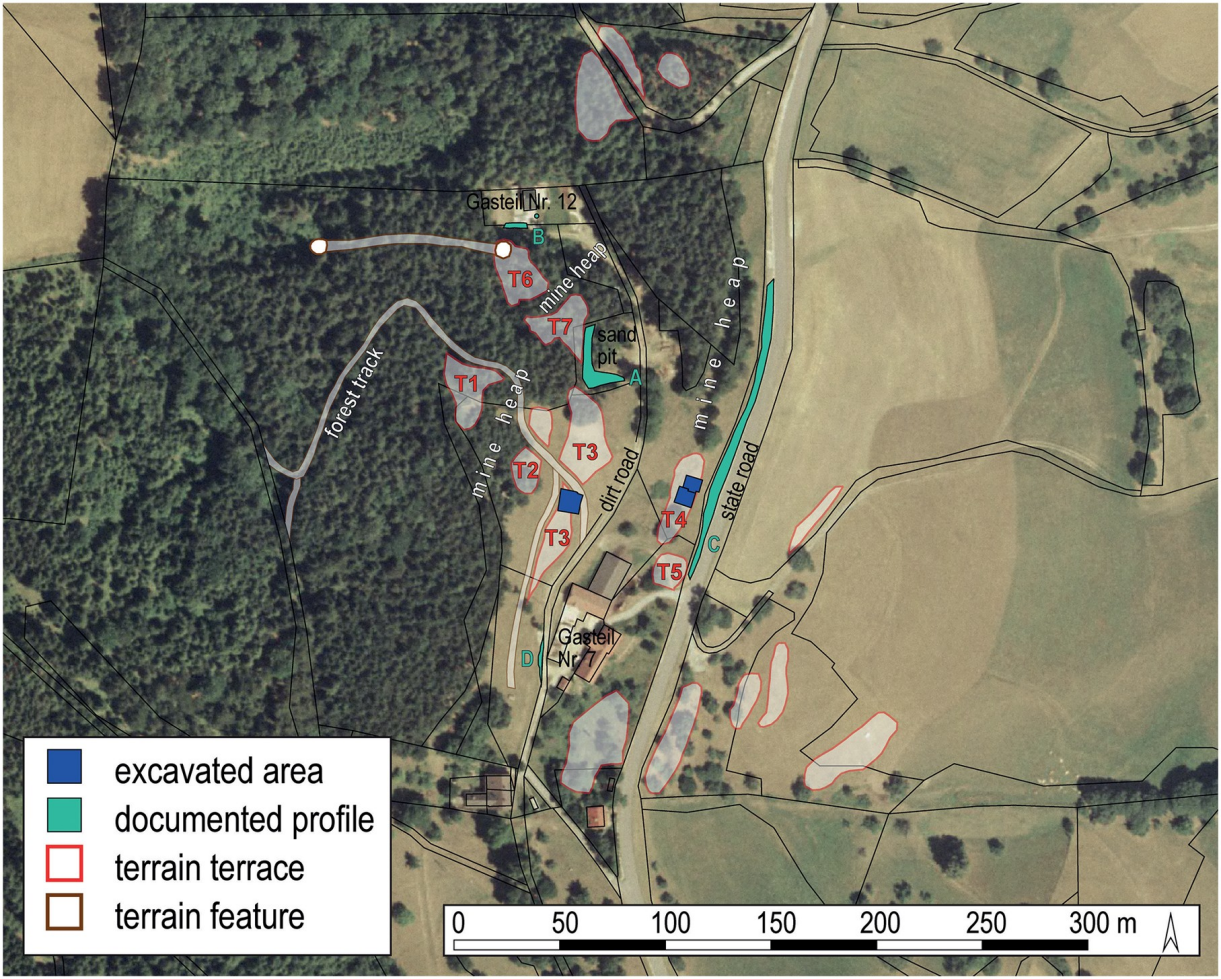
Prigglitz-Gasteil (Lower Austria). Plan of the Late Bronze Age site, showing visible terrain features and excavation areas on terraces T3 and T4. Aerial photograph: © Land Niederösterreich, with kind permission.

**Fig 3:**
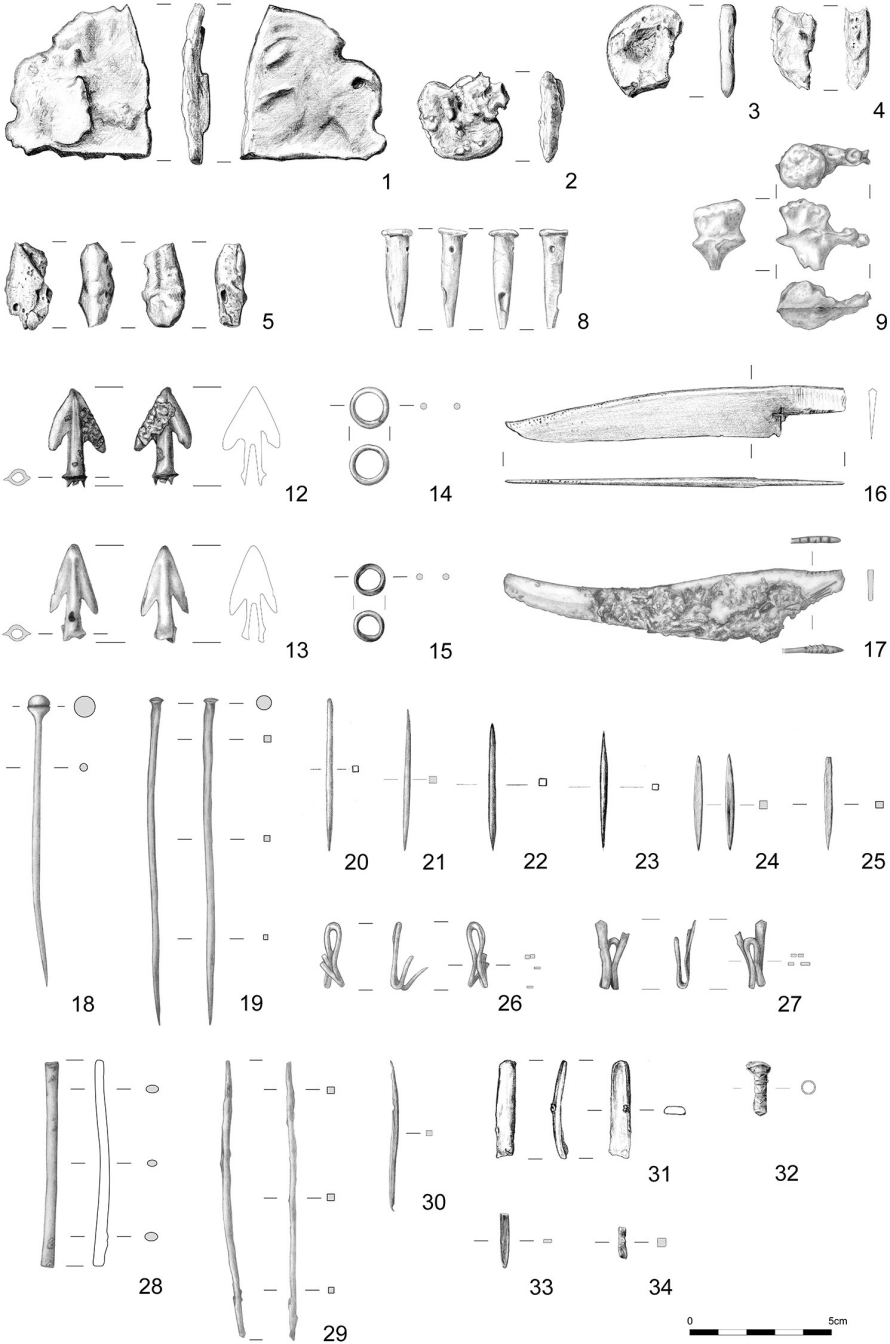
Prigglitz-Gasteil (Lower Austria). Analyses of select copper and copper alloy finds from the Late Bronze Age mining settlement (1–5 casting cakes; 8 casting cone; 9 casting sprue; 12–13 arrowheads; 14–15 rings; 16–17 knives; 18–19 dress pins; 20–25 awls; 26–27 belt clips; 28–29 bar fragments; 30 wire fragment; 31 bracelet fragment; 32 tubelet fragments; 33 tip fragment; 34 bar fragment). Scale 1:2 (drawings by Daniela Fehlmann and Ulrike Weinberger). The numbers correspond to those in the tables.

**Fig 4:**
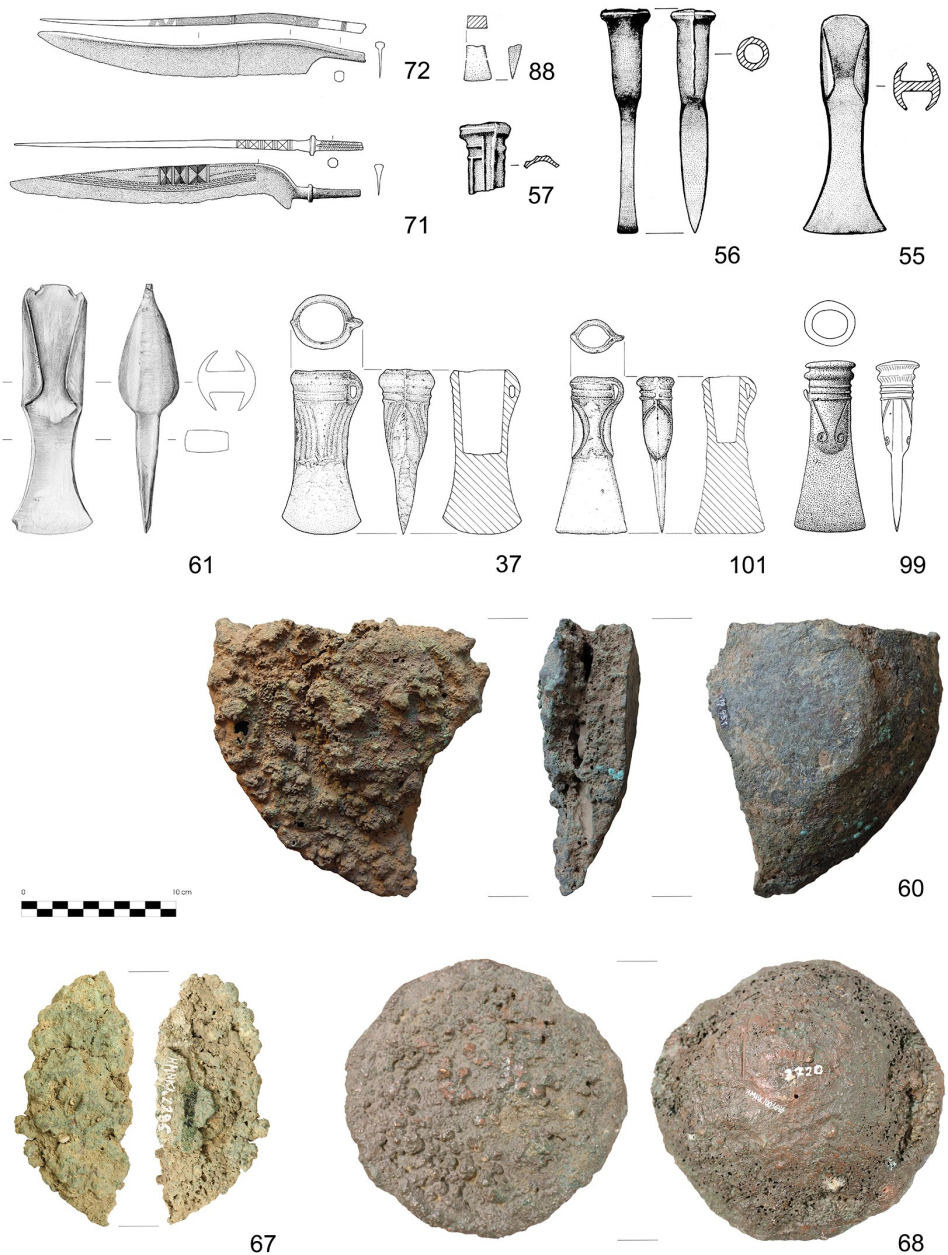
Selected copper and copper alloy finds from the Prigglitz-Gasteil surroundings. All sites are located in Lower Austria (see Fig 1). No 37: Prigglitz-Gasteil, Klausgraben; no 55, 56, 57, 60, 61: Grünbach-Gelände; no 67, 68: Neunkirchen-Klosteräcker; no 71, 72: Pottschach; no 88: Reichenau-Kammerwandgrotte; no 99: Sieding-Murrer; no 101: Ternitz-Gfieder. Scale 1:4 (drawings no 37, 88, 101: Franz Drost; no 55–57: Anton Distelberger; no 61: Daniela Fehlmann; no 71, 72, 99: unknown artist, reproduced from the publications quoted in Table 1; photographs: no 60: Michael Konrad; no 67, 68: Peter Trebsche). The numbers correspond to those in the tables.

The copper alloy and ore samples investigated in this paper were excavated from the Prigglitz-Gasteil site in the district of Neunkirchen, Lower Austria (Fig 1:Star). The site was discovered in the early 1950s and investigated in 1956 and 1958 by archaeologist Franz Hampl and mineralogist Robert Mayrhofer [25]. More than half a century later, Peter Trebsche resumed work at the site with geomagnetic surveys in 2010, archaeological excavations in 2010 to 2014, core drillings in 2013–2014 and 2017, and most recently, geoelectric and seismic prospections in 2017 and 2018 [16, 37–39]. These investigations showed a complete *chaîne opératoire* for copper metal production, including an opencast ore mine, beneficiation and primary ore smelting infrastructure, evidence for the refining of black copper, and alloying and casting of copper-based objects. All the above activities took place in approximately three hectares atop spoil heaps from the mine (Fig 2). The metal workshops, located on Terraces 3 and 4 at the site, where the bulk of the copper alloy finds discussed in this paper originated, date between the second half of the 11^th^ and the beginning of the 8^th^ centuries BC. The site’s absolute chronology is based on more than 75 radiocarbon samples of mostly short-lived charred plant remains and animal bones dating to the Late Urnfield Period (ca 1080–800 BC; [18]. From the 47 copper and copper alloy artefacts found during excavations from 2010–2014, 34 samples from Terraces 3 and 4 were chosen. The samples (Fig 3) represent each step in the metal-making process. They include seven small casting cakes, four casting residues (two casting drops, one casting cone, and one casting sprue), and 16 entirely preserved finished objects (six awls, two belt clips, two knives, two dress pins, two small rings, and two socketed arrowheads) and seven fragments of bronze objects (three bar fragments, one wire fragment, one bracelet fragment, one tubelet fragment, one tip fragment; cf. [16], Figs 7, 14, 19). Also included for analysis were a LBA socketed axe (Fig 4:37) and Late Neolithic copper flat axe from Klausgraben (Fig 1:5), located ~ 500 m away from the mining site. A fragment of a Bronze Age sewing needle and a spearhead fragment were also sampled from another findspot near the chapel at Gasteil (Fig 1:6).

This study includes finds from eight LBA sites to represent the overall regional distribution and exchange of metal materials (Fig 1). Objects from these sites were selected from museum collections where permission for sampling could be obtained (Fig 4). The first site, a cemetery from Pottschach (Fig 1:11), only 5 km away from Prigglitz-Gasteil, is of particular importance [24] since the same types and decoration of dress pins are found at both [37]. At Pottschach, several bronze objects (Fig 4:71 and 4:72) were discovered during railway construction work as early as 1840, and no individual grave contexts are available. The second site is an open lowland settlement 14 km away from Prigglitz at Urschendorf (Fig 1:17; [32] that was partially excavated in 2010. It was inhabited from the Late Urnfield Culture (Ha B) to Early Hallstatt period (Ha C) and could have been a consumer of the metal produced at Prigglitz-Gasteil. Four Late Urnfield and Early Hallstatt period objects were sampled from the site (three dress pins and one fragment of a pin). The third is the Kammerwandgrotte (Fig 1:2), located 7 km from the Prigglitz mine at Reichenau an der Rax. During excavations in 1998, evidence for Early and LBA activities, including metallurgy, was found in the waste heap immediately outside the cave [27]. Five copper alloy objects were analysed from the cave (one casting cake, two casting drops, one wire fragment, and one chisel fragment; Fig 4:88). These objects’ precise date has yet to be determined, but they likely belong to the Early or LBA. Based on the dates and proximity to Prigglitz, it is possible that raw copper from the mine was processed in the cave. Fourth is an important LBA hilltop settlement located at the Gelände in Grünbach am Schneeberg (Fig 1:8) located ~ 10 km from Prigglitz. Numerous metal artefacts and plate slag remnants were excavated from the plateau in 1935–1937, and there are concentrations of at least eight metal hoards in and around the settlement’s fort [22]. Only ten samples from two hoards were available for analysis, including one large casting cake (Fig 4:60), three small pieces of copper, three end-winged axes (Fig 4:55 and 4:61), one fragment of a socketed axe (Fig 4:57), one socketed chisel (Fig 4:56), one fragment of a bracelet, and one Late Neolithic hammer axe. Unfortunately, most of the artifacts from the fort were lost or are in private collections. There are also traces of mining activity within the region, but they have yet to be definitively dated [40]. Fifth, thirteen objects from a LBA hoard found in 1870 in the municipality of Ternitz at the village of Mahrersdorf (Fig 1:15) ~ 7.5 km from Prigglitz-Gasteil, were chosen for comparison to the above artifacts [21]. These objects’ chemical compositions, lead isotopic ratios, and metallographic character were studied previously [36]. The hoard contains, amongst other finds, a casting cake and a socketed pickaxe. Sixth, two hoards entirely composed of casting cakes found at Neunkirchen (Fig 1:16) and Heufeld near Gloggnitz (Fig 1:3) were also sampled. The hoard from Neunkirchen (11.5 km from Prigglitz) contained seven plano-convex ingots (Fig 4:68) and an irregular flat one (Fig 4:67) with a combined weight of ~ 40 kg, which, according to their shape and size, can be dated to the LBA [40]. Four of the plano-convex buns were sampled for this project. The other hoard, from Heufeld near Gloggnitz (~ 4.5 km from Prigglitz), comprises seven small irregularly shaped flat casting cakes with a weight range between ~ 105–215 g [41]. The cakes roughly date to the Bronze Age, and four out of seven of the cakes were sampled. Seventh, from the LBA mining region of Prein an der Rax (Fig 1:1), ~ 13 km from the Prigglitz mine, two finished copper-alloy objects (one dress pin and one awl), and four ore samples found during archaeological excavations in 1952–53 were used for comparison. Copper smelting activities in this region have been dated by radiocarbon to the Late Urnfield period (9th century BC; [18]. Last, three LBA single finds were used for comparison, including one socketed axe from the mountain Gfieder in Ternitz (Figs 1:12 and 4:101), one socketed spearhead from the mountain Gösing near Flatz (Fig 1:13), and one socketed axe from Sieding (Figs 1:7 and 4:99).

To further explore the diachronic development of copper production in the region, samples from two MBA (ca. 1650–1350 BC) graves excavated at Sieding (~ 4.5 km from Prigglitz; Fig 1:10) were analysed [29], 396–399 pl. 31). The studied materials included four *Lochhals* type dress pins, one fragment of a copper-alloy sheet belt, one small ring, and one small casting cake found near the burial mounds at the site. Another MBA dress pin, a stray find from nearby St. Johann (Fig 1:14), was also selected for analysis. Four Late Neolithic (4th millennium BC) finds near the Prigglitz mine were also sampled to provide a baseline characterisation of the copper used in the region during the Late Neolithic. These finds included one copper flat axe (type Altheim) from Gasteil-Klausgraben (Fig 1:5), one copper flat axe (type Altheim) from Gloggnitz-Semmeringtunnelportal (Fig 1:4), one fragment of a copper flat axe (type Vinča) from Kranichberg-Karlhöhe (Fig 1:9), and one hammer axe (type Şiria) from the Gelände hillfort near Grünbach am Schneeberg (Fig 1:8).

### 2.2 Methods

All analyses were carried out at the Curt-Engelhorn-Zentrum Archäometrie (CEZA) laboratory, Mannheim, Germany. All of the objects were sampled for chemical analysis, and a small selection for metallographic examination and their Pb isotopic ratios (metallographic analyses will be published elsewhere; see [42] (Table 1). X-ray fluorescence analyses of the objects were performed on drillings and the metallographic samples’ freshly polished surfaces. The drilling samples were analysed with an ARL Quant’ X (Thermo Fisher Scientific) XRF at 28 kV (with Pd filter) and 50 kV (with Cu filter); the polished samples were analysed using a Fischerscope X-ray XAN 150 (W-tube, 50 kV, Al-filter, 1 mm collimator, SD-detector, 50 s measuring time/spot, and 1–2 measurements/sample, depending on the sample size). Quantification and measurement of the XRF samples closely followed those described by [43]. The element concentrations were delineated as alloying with wt.% ≥ 1, trace ≤ 0.1, and not detected (n.d.). Sulfur (S) was not measured. All of the element concentrations in this paper are reported in wt.%, and non-destructive analytical techniques were used whenever possible. X-ray fluorescence and XRD analyses, which can be performed on untreated surfaces, were used on artefacts that could not be sampled due to their uniqueness or cultural importance. These analyses are also valuable indicators of any analytical offset between non-destructive and destructive techniques.

The ore samples were analysed using Neutron Activation Analyses (NAA). The ores were ground, and ~ 50 mg of the homogenized material was used for analysis. The sample material was irradiated with neutrons alongside suitable multi-element standards (NBS 400; BAM 227; BAM 376) for six hours in the TRIGA research reactor at the University of Mainz’s Institute for Nuclear Chemistry. The irradiation converts (activates) a small part of the sample’s elements into mostly short-lived radioactive nuclides; these nuclides’ decay produces gamma radiation specific to each element. The intensity of the radiation’s energy was measured with an HPGe Ortec spectrometer at the Curt-Engelhorn Centre for Archaeometry in Mannheim, and Ortec Gamma Vision software quantified the elemental concentrations; the detection limits were calculated based on the gamma spectra. To determine Pb and Bi (bismuth) content, 25 to 50 mg of sample powder was mixed with 3 ml of concentrated aqua regia and heated for 24 hours at 110°C in a Teflon^®^ beaker. The mixture was quantified using an iCAPQ Fisher Scientific Quadrupole Inductively Coupled Plasma—Mass Spectrometry (Q-ICP-MS) and calibrated with Certipur^®^ Multi-Element Standard XXI from Merck KGaA. A blank value is determined for each sample measurement, and detection limits were specified for each sample. Lead isotope analyses were carried out on a Thermo Scientific Neptune Plus High-resolution Multi-Collector Inductively Coupled Plasma—Mass Spectrometry (HR-MC-ICP-MS), following the procedure described by [44]. The samples were dissolved in nitric acid to separate the Pb from the matrix by ion chromatography. Then, for the measurements, an aliquot of the solution was separated and analysed via a Thermo Scientific iCAP 7200 Inductively Coupled Plasma—Optical Emission Spectrometer (ICP-OES).

## 3. Results

### 3.1 Chemical analyses

The XRF analyses on the metallographic samples did not consistently detect Bi, Mn, Co, Zn, Se, Cd, and Te (Table 2). However, these elements were detected in almost every drilling sample. While Te and Mn were always present at < 0.005%, < 0.002% Cd was detected in objects with Sn < 0.5% and all other drilling samples showed < 0.01% Cd. Selenium was detected at < 0.005% in all but three drilling samples, with three exceptions; axe no. 51 from Gloggnitz, pin no. 36 from Prigglitz-Gasteil, and socketed spearhead no. 50 from Flatz. In ~ 75% of the analysed finds, Co was found at < 0.001%. Higher amounts of Co, at up to 0.1%, were found in objects with tendentially higher amounts of Ni, As, Ag, and/or Sb with no obvious connection to typology or findspot. Bismuth concentrations were < 0.005% in most of the objects, with amounts > 0.02% usually correlated with Sb > 1%. The only exception to this relationship was a pin from Sieding (no. 96), which shows < 0.005% Bi and 1.4% Sb.

**Table 2:** Chemical analyses of the objects are given in wt.%; the chemical analyses of the copper ore samples are given in ppm (*italic*), despite for Cu and Fe. The chemical composition was analysed using NAA (ores) and XRF (all other objects). Tellurium was detected in all drilling samples of the objects at < 0.005 wt.%, but not in the metallographic ones. For some of the objects, both drillings and metallographic samples were analysed (nos. 60, 61, and 83).

No.	ID / Inv.no.	Find spot	Object	Cu	Mn	Fe	Co	Ni	Zn	As	Se	Ag	Cd	Sn	Sb	Pb	Bi	Te	Au	(Hg)
1	UF-22692.631	Prigglitz-Gasteil, site "Cu I"	casting cake**	**93.96**	< 0.005	5.81	0.06	0.07	< 0.1	0.03	< 0.005	0.00	< 0.002	**0.04**	0.03	< 0.005	< 0.005			
2	UF-10.432 (S073)	Prigglitz-Gasteil, site "Cu I"	casting cake***	**86.60**	0.03	2.08	< 0.01	0.19	< 0.1	0.04	< 0.005	0.07	< 0.01	**10.87**	0.11	0.01	< 0.005			
3	UF-10.862 (S074)	Prigglitz-Gasteil, site "Cu I"	casting cake	**94.65**	< 0.005	1.09	0.10	0.29	< 0.1	0.59	< 0.005	0.06	< 0.002	**0.05**	2.62	0.55	0.01			
4	UF-10.921 (S075)	Prigglitz-Gasteil, site "Cu I"	casting cake**	**98.94**	< 0.005	0.95	< 0.01	0.03	< 0.1	0.02	< 0.005	0.01	< 0.002	**0.01**	0.05	< 0.005	< 0.005			
5	UF-22692.675	Prigglitz-Gasteil, site "Cu I"	casting cake	**99.60**	< 0.005	0.11	0.01	0.06	0.07	0.01	< 0.005	0.01	< 0.002	**0.02**	0.09	0.01	< 0.005			
6	not inv. (GAST05)	Prigglitz-Gasteil, site "Cu I"	casting cake	**99.84**	< 0.005	< 0.05	< 0.01	0.14	< 0.1	0.01	< 0.005	0.00	< 0.002	**0.00**	0.01	< 0.005	< 0.005			
7	not inv. (GAST06)	Prigglitz-Gasteil, site "Cu I"	casting cake**	**98.69**	< 0.005	0.97	0.02	0.14	< 0.1	0.02	< 0.005	0.02	< 0.002	**0.01**	0.15	< 0.005	< 0.005			
8	not inv. (S068)	Prigglitz-Gasteil, site "Cu I"	casting cone	**91.33**	< 0.005	0.08	0.01	0.09	< 0.1	0.04	< 0.005	0.02	< 0.005	**8.04**	0.12	0.27	0.01			
9	UF-22692.187	Prigglitz-Gasteil, site "Cu I"	casting sprue	**88.88**	< 0.005	< 0.05	< 0.01	0.04	< 0.1	0.04	< 0.005	0.01	< 0.01	**10.90**	0.13	< 0.005	< 0.005			
10	UF-22692.1099	Prigglitz-Gasteil, site "Cu I"	casting drop	**88.68**	< 0.005	0.11	< 0.01	0.32	0.93	0.02	< 0.005	0.02	< 0.005	**9.67**	0.13	0.14	< 0.005			
11	UF-22692.2183	Prigglitz-Gasteil, site "Cu I"	casting drop	**96.68**	< 0.005	< 0.05	< 0.01	0.07	< 0.1	0.05	< 0.005	0.03	< 0.005	**2.95**	0.21	0.01	< 0.005			
12	UF-22692.10	Prigglitz-Gasteil, site "Cu I"	arrowhead (socketed)*,**	**84.68**	< 0.005	0.15	0.01	0.09	< 0.1	0.02	< 0.005	0.01	< 0.01	**14.92**	0.10	0.01	< 0.005			
13	UF-22692.792	Prigglitz-Gasteil, site "Cu I"	arrowhead (socketed)	**95.81**	< 0.005	0.09	0.02	0.18	< 0.1	0.06	< 0.005	0.06	< 0.005	**3.09**	0.61	0.02	0.05			
14	UF-22692.30	Prigglitz-Gasteil, site "Cu I"	ring	**90.30**	< 0.005	< 0.05	0.02	0.37	< 0.1	0.42	< 0.005	0.30	< 0.005	**7.80**	0.57	0.21	0.02			
15	UF-22692.789	Prigglitz-Gasteil, site "Cu I"	ring	**81.44**	< 0.005	0.07	0.02	0.79	< 0.1	1.00	< 0.005	0.48	< 0.01	**13.24**	1.54	1.40	0.02			
16	UF-10.964	Prigglitz-Gasteil, site "Cu I"	knife*,**	**83.74**	n.d.	0.73	n.d.	0.05	n.d.	n.d.	n.d.	n.d.	n.d.	**15.33**	n.d.	0.14	n.d.			
17	UF-22692.2188	Prigglitz-Gasteil, site "Cu I"	knife (type Griffangel)*,**	**89.20**	n.d.	0.19	n.d.	0.14	n.d.	0.14	n.d.	n.d.	n.d.	**9.91**	0.41	n.d.	n.d.			
18	UF-22692.913	Prigglitz-Gasteil, site "Cu I"	pin (type Kugelkopf)	**89.09**	< 0.005	0.53	0.01	0.06	0.14	0.02	< 0.005	0.01	< 0.01	**10.13**	0.00	0.02	< 0.005			
19	UF-22692.1853	Prigglitz-Gasteil, site "Cu I"	pin (type Nagelkopf)	**90.48**	< 0.005	< 0.05	< 0.01	0.06	< 0.1	< 0.01	< 0.005	0.02	< 0.005	**8.52**	0.05	0.86	0.01			
20	UF-22692.881	Prigglitz-Gasteil, site "Cu I"	awl	**96.57**	< 0.005	0.06	< 0.01	0.12	< 0.1	0.04	< 0.005	0.09	< 0.005	**2.89**	0.19	0.03	0.01			
21	UF-22692.1140A	Prigglitz-Gasteil, site "Cu I"	awl*	**90.35**	n.d.	n.d.	n.d.	n.d.	n.d.	n.d.	n.d.	n.d.	n.d.	**9.53**	0.12	n.d.	n.d.			
22	UF-22692.1272	Prigglitz-Gasteil, site "Cu I"	awl*	**89.02**	n.d.	n.d.	n.d.	n.d.	n.d.	n.d.	n.d.	n.d.	n.d.	**10.98**	n.d.	n.d.	n.d.			
23	UF-22692.1672	Prigglitz-Gasteil, site "Cu I"	awl*	**86.02**	n.d.	n.d.	n.d.	0.07	n.d.	n.d.	n.d.	n.d.	n.d.	**13.73**	0.10	0.08	n.d.			
24	UF-22692.413	Prigglitz-Gasteil, site "Cu I"	awl	**87.53**	< 0.005	0.24	0.01	0.37	6.54	< 0.01	< 0.005	0.01	< 0.005	**4.34**	0.12	0.84	0.01			
25	UF-22692.666	Prigglitz-Gasteil, site "Cu I"	awl	**85.26**	< 0.005	0.13	< 0.01	1.21	4.89	0.01	< 0.005	0.01	< 0.005	**7.81**	0.08	0.59	0.01			
26	UF-22692.1673	Prigglitz-Gasteil, site "Cu I"	clip (belt)*,**	**88.00**	n.d.	0.06	n.d.	0.28	n.d.	0.32	n.d.	0.28	n.d.	**10.34**	0.37	0.34	n.d.			
27	UF-22692.1652	Prigglitz-Gasteil, site "Cu I"	clip (belt)*	**91.15**	n.d.	0.22	n.d.	0.07	n.d.	0.03	n.d.	n.d.	n.d.	**8.29**	0.24	n.d.	n.d.			
28	UF-22692.912	Prigglitz-Gasteil, site "Cu I"	bar (fragment)*	**87.44**	n.d.	n.d.	n.d.	0.03	n.d.	n.d.	n.d.	n.d.	n.d.	**11.85**	0.06	0.61	n.d.			
29	UF-22692.1854	Prigglitz-Gasteil, site "Cu I"	bar (fragment)	**90.34**	< 0.005	0.68	0.02	0.11	< 0.1	0.04	< 0.005	0.01	< 0.005	**8.53**	0.27	0.01	0.01			
30	UF-22692.303	Prigglitz-Gasteil, site "Cu I"	wire (fragment)	**89.62**	< 0.005	< 0.05	< 0.01	0.08	< 0.1	0.05	< 0.005	0.02	< 0.01	**10.00**	0.08	0.16	0.01			
31	UF-22692.1780	Prigglitz-Gasteil, site "Cu I"	bracelet (fragment)*	**86.85**	n.d.	0.13	n.d.	0.04	n.d.	n.d.	n.d.	n.d.	n.d.	**12.85**	0.13	n.d.	n.d.			
32	UF-22692.1110	Prigglitz-Gasteil, site "Cu I"	tubelet (fragment)	**88.62**	< 0.005	0.55	< 0.01	0.29	1.36	0.04	< 0.005	0.01	< 0.005	**8.90**	0.07	0.17	< 0.005			
33	UF-22692.2177	Prigglitz-Gasteil, site "Cu I"	tip (fragment)**	**83.45**	n.d.	0.25	n.d.	0.04	n.d.	0.14	n.d.	n.d.	n.d.	**15.76**	0.31	n.d.	0.06			
34	UF-22692.2335	Prigglitz-Gasteil, site "Cu I"	bar (fragment)	**73.52**	< 0.005	0.34	< 0.01	0.47	17.96	0.03	< 0.005	0.01	< 0.005	**5.11**	0.16	2.38	0.02			
35	S005	Prigglitz-Gasteil, Kapelle	spear head	**87.26**	< 0.005	0.09	0.02	0.51	< 0.1	0.28	< 0.005	0.03	< 0.01	**11.17**	0.28	0.34	0.01			
36	S021	Prigglitz-Gasteil, Kapelle	pin	**97.41**	< 0.005	< 0.05	< 0.01	0.01	1.57	0.04	0.01	0.16	< 0.005	**0.50**	0.01	0.27	0.01			
37	S001	Prigglitz-Gasteil, Klausgraben	axe (socketed)	**91.23**	< 0.005	< 0.05	0.02	0.12	< 0.1	0.02	< 0.005	0.01	< 0.005	**8.51**	0.09	0.01	< 0.005			
38	S044	Bürg near Prigglitz-Gasteil, Klausgraben	axe (flat)	**99.03**	< 0.005	< 0.05	< 0.01	0.02	< 0.1	0.89	< 0.005	0.01	< 0.002	**0.01**	0.01	0.03	< 0.005			
39	UF-22692.730	Prigglitz-Gasteil, site "Cu I"	copper ore	**18.30**	*n*.*d*.	29.00	*13*.*70*	*90*.*00*	*44*.*00*	*89*.*00*	*2*.*70*	*10*.*20*	*n*.*d*.	*100*.*00*	*105*.*30*	*11*.*20*	*6*.*27*	*< 18*	*0*.*38*	*27*.*00*
40	UF-22692.892	Prigglitz-Gasteil, site "Cu I"	copper ore	**31.40**	*n*.*d*.	58.00	*210*.*00*	*230*.*00*	*30*.*00*	*393*.*00*	*7*.*70*	*19*.*00*	*n*.*d*.	*< 1000*	*223*.*00*	*3*.*19*	*0*.*46*	*23*.*00*	*1*.*34*	*44*.*00*
41	UF-22692.1142	Prigglitz-Gasteil, site "Cu I"	copper ore	**7.65**	*n*.*d*.	< 8	*2*.*00*	*< 160*	*19*.*00*	*30*.*50*	*0*.*70*	*5*.*10*	*n*.*d*.	*< 160*	*81*.*40*	*8*.*92*	*0*.*46*	*< 15*	*0*.*19*	*17*.*00*
42	UF-22692.2514	Prigglitz-Gasteil, site "Cu I"	copper ore	**6.93**	*n*.*d*.	< 5	*22*.*00*	*< 240*	*398*.*00*	*940*.*00*	*< 4*	*48*.*00*	*n*.*d*.	*100*.*00*	*2075*.*00*	*6*.*99*	*42*.*30*	*17*.*00*	*0*.*2*	*n*.*b*.
43	UF-22692.2553	Prigglitz-Gasteil, site "Cu I"	copper ore	**2.71**	*n*.*d*.	2.60	*6*.*00*	*46*.*00*	*127*.*00*	*211*.*00*	*< 3*	*4*.*60*	*n*.*d*.	*< 360*	*607*.*00*	*0*.*87*	*4*.*26*	*14*.*00*	*0*.*033*	*n*.*b*.
44	UF-22692.2558	Prigglitz-Gasteil, site "Cu I"	copper ore	**1.67**	*n*.*d*.	< 3	*5*.*10*	*37*.*00*	*35*.*00*	*77*.*90*	*2*.*20*	*2*.*70*	*n*.*d*.	*< 250*	*301*.*00*	*0*.*82*	*2*.*74*	*< 20*	*0*.*024*	*25*.*00*
45	UF-22692.2666	Prigglitz-Gasteil, site "Cu I"	copper ore	**6.09**	*n*.*d*.	4.30	*16*.*80*	*71*.*00*	*41*.*00*	*66*.*00*	*7*.*00*	*4*.*80*	*n*.*d*.	*< 360*	*332*.*00*	*8*.*28*	*2*.*13*	*10*.*00*	*0*.*1*	*42*.*00*
46	UF-22692.2678	Prigglitz-Gasteil, site "Cu I"	copper ore	**8.54**	*n*.*d*.	4.30	*0*.*55*	*< 93*	*14*.*00*	*9*.*20*	*< 1*	*2*.*20*	*n*.*d*.	*< 130*	*32*.*90*	*1*.*69*	*1*.*71*	*7*.*00*	*0*.*086*	*n*.*b*.
47	UF-22692.2682	Prigglitz-Gasteil, site "Cu I"	copper ore	**1.94**	*n*.*d*.	< 2	*0*.*86*	*33*.*00*	*18*.*00*	*5*.*00*	*< 1*	*0*.*90*	*n*.*d*.	*< 49*	*17*.*48*	*0*.*51*	*0*.*32*	*< 11*	*0*.*056*	*n*.*b*.
48	UF-10796.2	Prigglitz-Gasteil, site "Cu I"	copper ore	**16.60**	*n*.*d*.	< 11	*0*.*91*	*48*.*00*	*10*.*00*	*28*.*60*	*6*.*20*	*11*.*00*	*n*.*d*.	*80*.*00*	*76*.*60*	*12*.*06*	*152*.*33*	*< 19*	*0*.*24*	*21*.*00*
49	UF-22692.1150	Prigglitz-Gasteil, site "Cu I"	copper ore	**12.05**	*n*.*d*.	< 9	*11*.*80*	*31*.*00*	*633*.*00*	*30*.*80*	*< 2*	*2*.*20*	*n*.*d*.	*< 92*	*90*.*60*	*0*.*59*	*0*.*25*	*13*.*00*	*0*.*154*	*n*.*b*.
50	S025	Flatz-Gösing	spear head	**84.03**	< 0.005	0.30	0.12	0.79	< 0.1	0.96	< 0.01	0.40	< 0.005	**9.11**	1.06	3.20	0.04			
51	S002	Gloggnitz-Semmeringtunnelportal	axe (flat)	**99.72**	< 0.005	0.14	< 0.01	0.03	< 0.1	0.01	0.01	0.05	< 0.002	**0.02**	0.01	0.01	< 0.005			
52	S014	Grünbach-Gelände	copper fragment**	**89.32**	< 0.005	0.57	0.04	0.31	0.07	0.44	< 0.005	0.16	< 0.005	**7.92**	0.28	0.86	0.03			
53	S015	Grünbach-Gelände	copper fragment**	**97.31**	< 0.005	0.80	0.03	0.03	0.29	0.37	< 0.005	0.08	< 0.002	**0.11**	0.07	0.88	0.04			
54	S016	Grünbach-Gelände	copper fragment	**98.81**	< 0.005	0.49	< 0.01	0.07	< 0.1	0.56	< 0.005	0.01	< 0.002	**0.01**	0.01	0.04	< 0.005			
55	S049	Grünbach-Gelände	axe (end-winged)**	**94.39**	< 0.01	< 0.05	0.02	0.53	< 0.1	0.72	< 0.01	0.07	< 0.005	**3.87**	0.29	0.09	0.01			
56	S050	Grünbach-Gelände	chisel (socketed)**	**91.88**	< 0.005	0.06	0.02	0.55	< 0.1	0.84	< 0.005	0.20	< 0.005	**5.47**	0.59	0.37	0.02			
57	S051	Grünbach-Gelände	axe (socketed)**	**92.75**	< 0.005	0.24	0.03	0.24	0.12	0.64	< 0.005	0.18	< 0.005	**4.90**	0.41	0.46	0.03			
58	S070	Grünbach-Gelände	axe (hammer-shaped)	**99.99**	< 0.005	< 0.05	< 0.01	< 0.1	< 0.1	< 0.005	< 0.005	< 0.002	< 0.002	**0.00**	< 0.002	0.01	< 0.005			
59	S081	Grünbach-Gelände	bracelet	**91.84**	< 0.005	0.17	0.03	0.34	< 0.1	0.61	< 0.005	0.31	< 0.005	**5.42**	0.88	0.35	0.04			
60a	UF-19451	Grünbach-Gelände	casting cake**	**98.80**	< 0.005	0.55	0.04	0.17	< 0.1	0.32	< 0.005	0.06	< 0.002	**0.01**	0.03	0.01	< 0.005			
60b	UF-19451	Grünbach-Gelände	casting cake**	**98.55**	< 0.005	0.68	0.05	0.17	< 0.1	0.38	< 0.005	0.06	< 0.002	**0.06**	0.04	0.01	< 0.005			
61a	UF-19453	Grünbach-Gelände	axe (end-winged)	**90.29**	< 0.005	0.12	0.04	0.41	0.13	0.63	< 0.005	0.16	< 0.005	**7.57**	0.41	0.23	0.01			
61b	UF-19453	Grünbach-Gelände	axe (end-winged)*	**89.04**	n.d.	0.09	n.d.	0.40	n.d.	0.51	n.d.	0.15	n.d.	**9.10**	0.52	0.19	n.d.			
62	H001	Heufeld	casting cake	**97.69**	< 0.005	1.74	0.01	0.09	< 0.1	0.08	< 0.005	0.01	< 0.002	**0.04**	0.35	< 0.005	< 0.005			
63	H003	Heufeld	casting cake	**97.84**	< 0.005	1.69	0.01	0.08	< 0.1	0.11	< 0.005	0.01	< 0.002	**0.03**	0.23	0.01	< 0.005			
64	H004	Heufeld	casting cake	**97.74**	< 0.005	1.61	0.01	0.10	< 0.1	0.11	< 0.005	0.01	< 0.002	**0.04**	0.38	0.01	< 0.005			
65	H005	Heufeld	casting cake	**99.21**	0.01	0.65	< 0.01	0.05	< 0.1	0.02	< 0.005	0.00	< 0.002	**0.01**	0.06	< 0.005	< 0.005			
66	S071	Kranichberg-Karlhöhe	axe (fragment)	**97.84**	< 0.005	< 0.05	< 0.01	0.37	< 0.1	1.72	< 0.005	0.03	< 0.002	**0.01**	0.02	0.01	< 0.005			
67	3720 (S067)	Neunkirchen-Klosteräcker	casting cake	**99.78**	< 0.005	0.10	< 0.01	0.01	< 0.1	0.01	< 0.005	0.01	< 0.002	**0.02**	0.07	0.01	< 0.005			
68	9024 (S060)	Neunkirchen-Klosteräcker	casting cake**	**98.19**	0.03	1.58	< 0.01	< 0.1	< 0.1	0.03	< 0.005	0.02	< 0.002	**0.08**	0.05	0.02	< 0.005			
69	9027 (S063)	Neunkirchen-Klosteräcker	casting cake**	**98.85**	0.01	0.97	< 0.01	0.04	< 0.1	0.03	< 0.005	0.01	< 0.002	**0.01**	0.08	0.00	< 0.005			
70	9029 (S065)	Neunkirchen-Klosteräcker	casting cake	**99.27**	< 0.005	0.60	< 0.01	0.03	< 0.1	0.01	< 0.005	0.01	< 0.002	**0.00**	0.07	0.00	< 0.005			
71	72.484	Pottschach	knife	**89.05**	< 0.005	< 0.05	0.01	0.07	< 0.1	0.02	< 0.005	0.02	< 0.01	**10.59**	0.21	0.02	< 0.005			
72	72.485	Pottschach	knife	**88.92**	< 0.005	0.36	0.01	0.06	< 0.1	0.13	< 0.005	0.09	< 0.005	**9.17**	1.24	0.02	0.01			
73	72.487 A	Pottschach	pin (type Vasenkopf)	**91.43**	< 0.005	0.24	< 0.01	0.07	< 0.1	0.04	< 0.005	0.01	< 0.005	**7.95**	0.16	0.10	< 0.005			
74	72.487 B	Pottschach	pin (type Vasenkopf)	**90.24**	< 0.005	0.28	0.01	0.08	< 0.1	0.04	< 0.005	0.01	< 0.005	**8.84**	0.20	0.30	< 0.005			
75	72.488	Pottschach	pin (type Vasenkopf)	**90.43**	< 0.005	0.10	0.01	0.07	< 0.1	0.01	< 0.005	0.02	< 0.005	**9.21**	0.13	0.01	< 0.005			
76	72.489	Pottschach	bracelet	**90.35**	< 0.005	< 0.05	< 0.01	< 0.1	< 0.1	< 0.005	< 0.005	0.00	< 0.005	**9.63**	0.01	0.01	< 0.005			
77	72.491 A	Pottschach	bracelet	**89.64**	< 0.005	< 0.05	< 0.01	0.08	< 0.1	0.01	< 0.005	0.01	< 0.01	**10.17**	0.08	0.01	< 0.005			
78	72.491 B	Pottschach	bracelet	**88.38**	< 0.005	< 0.05	< 0.01	0.03	< 0.1	0.01	< 0.005	0.01	< 0.01	**11.41**	0.07	0.09	< 0.005			
79	72.491 C	Pottschach	bracelet	**87.52**	< 0.005	0.05	< 0.01	0.09	< 0.1	0.03	< 0.005	0.02	< 0.01	**12.04**	0.24	0.01	< 0.005			
80	72.492 A	Pottschach	bracelet	**94.86**	< 0.005	0.12	0.01	0.06	< 0.1	0.01	< 0.005	0.01	< 0.005	**4.81**	0.06	0.06	< 0.005			
81	72.492 B	Pottschach	bracelet	**91.31**	< 0.005	< 0.05	< 0.01	0.02	< 0.1	0.03	< 0.005	0.02	< 0.005	**8.40**	0.06	< 0.005	0.17			
82	UF-9751 (S078)	Prein, site III	pin (type Rippenkopf)	**88.19**	< 0.005	0.15	< 0.01	0.04	< 0.1	0.02	< 0.005	0.01	< 0.01	**11.51**	0.09	< 0.005	< 0.005			
83	UF-9958	Prein, site V	awl*	**93.89**	n.d.	0.08	n.d.	0.05	n.d.	n.d.	n.d.	n.d.	n.d.	**5.19**	0.10	0.69	n.d.			
84	UF-9723.1	Prein, site II	copper ore	**7.16**	*n*.*d*.	< 3	*13*.*50*	*43*.*00*	*332*.*00*	*1470*.*00*	*2*.*50*	*7*.*30*	*n*.*d*.	*< 330*	*550*.*00*	*5*.*45*	*106*.*56*	*14*.*00*	*2*.*75*	*590*.*00*
85	UF-9723.2	Prein, site II	copper ore	**3.08**	*n*.*d*.	1.40	*6*.*60*	*60*.*00*	*34*.*00*	*274*.*00*	*2*.*10*	*2*.*30*	*n*.*d*.	*80*.*00*	*207*.*00*	*0*.*52*	*10*.*56*	*9*.*00*	*9*.*6*	*189*.*00*
86	UF-9722a.1	Prein, site II	copper ore	**1.11**	*n*.*d*.	0.90	*3*.*70*	*34*.*00*	*34*.*00*	*244*.*00*	*0*.*80*	*3*.*80*	*n*.*d*.	*26*.*00*	*114*.*70*	*0*.*66*	*56*.*59*	*< 140*	*0*.*6*	*146*.*00*
87	UF-9970	Prein, site VII	copper ore	**1.13**	*n*.*d*.	20.00	*43*.*00*	*130*.*00*	*71*.*00*	*33*.*50*	*1*.*50*	*4*.*50*	*n*.*d*.	*< 170*	*19*.*90*	*0*.*74*	*0*.*90*	*< 26*	*< 0*,*03*	*< 2*
88a	not inv. (Fn. 24, S004, S041)	Reichenau-Kammerwandgrotte	chisel	**91.81**	< 0.005	< 0.05	0.05	0.15	< 0.1	0.15	< 0.005	0.06	< 0.005	**7.06**	0.23	0.48	0.01			
88b	not inv. (Fn. 24, S004, S041)	Reichenau-Kammerwandgrotte	chisel*	**91.40**	n.d.	n.d.	n.d.	0.15	n.d.	0.19	n.d.	0.08	n.d.	**7.54**	0.25	0.39	n.d.			
89	not inv. (S010)	Reichenau-Kammerwandgrotte	casting residue	**99.64**	< 0.005	0.21	< 0.01	0.08	< 0.1	0.01	< 0.005	0.00	< 0.002	**0.03**	0.02	< 0.005	< 0.005			
90	not inv. (S011)	Reichenau-Kammerwandgrotte	casting residue	**99.83**	< 0.005	0.11	< 0.01	0.02	< 0.1	0.01	< 0.005	0.00	< 0.002	**0.01**	0.02	< 0.005	< 0.005			
91	not inv. (S013)	Reichenau-Kammerwandgrotte	wire*	**87.54**	n.d.	0.14	n.d.	0.06	n.d.	0.06	n.d.	n.d.	n.d.	**11.96**	0.20	0.05	n.d.			
92	not inv. (S069)	Reichenau-Kammerwandgrotte	casting cake	**99.46**	< 0.005	0.41	< 0.01	0.04	< 0.1	0.02	< 0.005	0.01	< 0.002	**0.02**	0.05	< 0.005	< 0.005			
93	UF-4553 (S033)	Sieding-Hügel 1	belt*,**	**82.01**	n.d.	0.25	n.d.	0.58	n.d.	1.81	n.d.	n.d.	n.d.	**13.98**	0.53	0.85	n.d.			
94	UF-4554 (S036)	Sieding-Hügel 1	pin (type Lochhals)	**87.62**	< 0.005	0.15	0.01	0.28	< 0.1	0.46	< 0.005	0.01	< 0.01	**11.08**	0.34	0.05	< 0.005			
95	UF-4555 (S037)	Sieding-Hügel 1	pin (type Lochhals)	**88.39**	< 0.005	0.11	0.02	0.37	< 0.1	0.57	< 0.005	0.02	< 0.01	**10.09**	0.41	0.03	< 0.005			
96	UF-4957	Sieding-Hügel 1	pin (type Lochhals)	**92.29**	< 0.005	0.26	0.02	0.34	< 0.1	0.61	< 0.005	0.03	< 0.005	**5.02**	1.39	0.03	< 0.005			
97	UF-4958	Sieding-Hügel 1	pin (type Lochhals)	**94.30**	< 0.005	0.51	0.02	0.22	< 0.1	0.32	< 0.005	0.01	< 0.005	**4.08**	0.44	0.11	< 0.005			
98	UF-4959 (S012)	Sieding-Hügel 1	ring	**86.38**	< 0.005	0.18	0.11	0.71	0.07	0.59	< 0.005	0.47	< 0.005	**9.00**	1.32	1.17	0.02			
99a	UF-5098	Sieding-Murrer	axe (socketed)	**91.79**	< 0.005	0.11	0.01	0.13	< 0.1	0.05	< 0.005	0.01	< 0.005	**7.70**	0.09	0.10	< 0.005			
99b	UF-5098	Sieding-Murrer	axe (socketed)*	**89.17**	n.d.	0.11	n.d.	0.11	n.d.	0.06	n.d.	n.d.	n.d.	**10.32**	0.14	0.09	n.d.			
100	UF-6117 (S023)	Sieding-Steinparzkreuz	casting cake	**89.56**	< 0.005	5.38	0.14	0.47	< 0.1	3.94	< 0.005	0.03	< 0.002	**0.19**	0.29	< 0.005	< 0.005			
101	not inv. (S003)	Ternitz-Gfieder	axe (socketed)	**91.09**	< 0.005	0.16	< 0.01	0.06	< 0.1	0.01	< 0.005	0.00	< 0.005	**8.65**	0.02	< 0.005	< 0.005			
102	UF-9019 (S076)	Ternitz-St. Johann	pin	**88.99**	< 0.005	0.27	0.02	0.30	< 0.1	0.62	< 0.005	0.01	< 0.005	**9.48**	0.29	0.02	< 0.005			
103	not inv. (Fn. 1, S008)	Urschendorf	pin (type Vasenkopf)	**91.80**	< 0.005	0.10	< 0.01	0.08	< 0.1	0.10	< 0.005	0.03	< 0.005	**7.35**	0.41	0.13	< 0.005			
104	not inv. (Fn. 1, S009)	Urschendorf	tip (fragment)	**88.93**	< 0.005	0.27	0.07	0.33	< 0.1	1.16	< 0.005	0.08	< 0.005	**8.36**	0.32	0.45	0.04			
105	not inv. (Fn. 32, S007)	Urschendorf	pin (type Zylinderkopf)	**89.40**	< 0.005	0.15	0.04	0.94	0.10	0.73	< 0.005	0.04	< 0.005	**8.12**	0.37	0.11	0.00			
106	not inv. (Fn. 7, S006)	Urschendorf	pin (type Rollenkopf)	**88.38**	< 0.005	0.07	0.04	0.45	< 0.1	0.89	< 0.005	0.62	< 0.005	**6.69**	1.09	1.75	0.03			

*analyses made on metallographic samples

**presence of corrosion and low amount of material for analyses

***corrosion products and almost no metal was present.

Zinc was detected at < 0.01% in all but five objects from Prigglitz. One casting drop (no. 10) with 9.7% Sn also contains ~ 0.9% Zn, suggesting the mixing of different metal types took place at the site. A tubelet fragment at the site (no. 32) also contains ~ 1.4% Zn and 9% Sn. Awls no. 24 and 25 contain significant amounts of Zn and some Sn. The former contains 6.5% Zn and 4.3% Sn, and the latter 4.9% Zn and 7.8% Sn. Pin no. 36, a surface find, with 1.6% Zn and ~ 0.5% Sn, has an uncertain date but is nevertheless mentioned here due to its chemical content.

Only 11 objects show notable amounts of Pb greater than 0.5%. Most of them derive from Prigglitz and include: awls no. 24 and 25; pin, no. 19; bar, no. 28; ring, no. 15; and bar fragment no. 34. In addition to the objects, casting cake no. 3 contains 0.55% Pb. The other objects containing notable amounts of Pb include an awl from Prein an der Rax (no.83, 0.7%), a ring from Sieding (no.98, 1.2%), a pin from Urschendorf-St. Egyden (no. 106, 1.7%), and a socketed spearhead from Flatz (no. 50, 3.2%).

More than half of the objects contain < 0.2% Fe and another 18 < 0.5%. Seven of the objects contain 0.5–1%, including pins from Sieding (no. 97) and Prigglitz (nos. 18, 29), a tubelet from Prigglitz (no. 32), and casting cakes from Heufeld (no. 65) and Neunkirchen (no. 70). All the objects with more than 1%, and up to 5.4%, Fe, are casting cakes from Prigglitz, Heufeld, and Sieding. Three casting cakes from Heufeld consistently contain over 1%, suggesting that they originated from the same source or production process.

Casting cakes no. 3 from Prigglitz and no. 100 from Sieding have 0.6% and 3.9% As, respectively. The former also contains 2.6% Sb. Higher concentrations of As, > 0.5%, correlate in most cases with higher amounts of Ag (> 0.1%) and Sb (> 0.5%). Of note, the casting cake from Sieding contains almost 4% As and over 5% Fe, which is unique in the assemblage. Three finds from Grünbach, two Freudenberg-type axes and one piece of copper, contain ~ 0.5–0.6% As, as do half of those from Sieding.

Half of the objects have < 0.2% Sb, 20 contain over 0.5%, and included in the total is one casting cake (Prigglitz, no. 3). Higher concentrations of Sb, at over 0.5%, correlate with low Fe (< 0.4%) and higher amounts of Ag (0.1–0.6%). An exception to this trend is casting cake no. 3 from Prigglitz. Only two objects, with > 0.1% Ag, also have < 0.4% Sb (pin, Prigglitz, no. 36; axe, Grünbach, no. 55). Antimony in amounts > 0.5% is also often correlated with higher As (0.5–1%). Exceptions to this trend are a Late Neolithic axe from Bürg near Prigglitz-Gasteil (no. 38), a piece of copper from Grünbach (no. 54), and a Late Neolithic flat axe from Kranichberg (no. 66) that contains 0.6–1.7% As and < 0.02% Sb.

Nickel concentrations are usually < 0.5 wt.% in the casting cakes and unfinished copper objects. More than half of the objects have < 0.1, and 7 of them, with 0.5–1.2, do not show any typological, chronological, or geographical association. However, three of them, a ring from Sieding (no. 98), a ring from Prigglitz (no. 15), and a socketed spearhead from Flatz (no. 50), have 0.6–1% As, ~ 0.5% Ag, and 1–1.5% Sb, respectively. Only nine objects have more than 0.1% Ag, which is usually present with higher Sb (0.5–2.4%) and As (0.5–1%). The only exception to this trend is pin no. 36 from Prigglitz.

All the casting cakes, copper fragments, and Late Neolithic axes (flat axe, no. 38, Bürg; flat axe, no. 51, Gloggnitz; hammer-shaped axe, no. 58, Grünbach; flat axe, no. 66, Kranichberg) contain no Sn or have concentrations < 0.2%. Of note, however, is pin no. 36 from Prigglitz, which contains ~ 0.5% Sn and 0.16% Ag. Objects containing more Sn, between 3–6 wt.%, do not correlate with type or findspot. Of interest, one socketed arrowhead from Prigglitz, no. 12, contains nearly 15 wt.% Sn while another specimen of the same type (no. 13) contains only ~ 3 wt.%.

### 3.2 Lead isotope analyses

Lead isotope analyses were performed on twenty-nine copper and copper alloy objects. Included in these analyses are objects from Prigglitz-Gasteil and findspots surrounding the site. Five copper ores from Prigglitz and Prein an der Rax were also analysed; however, 10 out of 15 samples did not contain sufficient Pb for quantification. The results of these analyses are given in Table 3.

**Table 3:** Lead isotope ratios for the materials studied.

No.	ID / Inv.no.	site	object	^208^Pb/^206^Pb	2σ	^207^Pb/^206^Pb	2σ	^206^Pb/^204^Pb	2σ	^208^Pb/^204^Pb	2σ	^207^Pb/^204^Pb	2σ
3	UF-10.862 (S074)	Prigglitz-Gasteil, site "Cu I"	casting cake	2.0996	0.0001	0.85288	0.00002	18.364	0.001	38.558	0.013	15.663	0.001
5	UF-22692.675	Prigglitz-Gasteil, site "Cu I"	casting cake	2.0771	0.0001	0.84112	0.00002	18.614	0.002	38.662	0.005	15.656	0.001
7	not inv. (GAST06)	Prigglitz-Gasteil, site "Cu I"	casting cake*	2.0212	0.0001	0.80828	0.00001	19.437	0.001	39.286	0.005	15.711	0.001
12	UF-22692.10	Prigglitz-Gasteil, site "Cu I"	arrowhead (socketed)	2.0792	0.0001	0.84373	0.00002	18.584	0.001	38.64	0.003	15.68	0.001
13	UF-22692.792	Prigglitz-Gasteil, site "Cu I"	arrowhead (socketed)	2.1023	0.0001	0.85389	0.00002	18.351	0.003	38.579	0.016	15.67	0.003
15	UF-22692.789	Prigglitz-Gasteil, site "Cu I"	ring	2.0981	0.0001	0.85201	0.00001	18.384	0.001	38.57	0.004	15.663	0.001
18	UF-22692.913	Prigglitz-Gasteil, site "Cu I"	pin (type Kugelkopf)*	2.097	0.0001	0.85452	0.00001	18.301	0.001	38.376	0.006	15.638	0.001
23	UF-22692.1672	Prigglitz-Gasteil, site "Cu I"	awl	2.1028	0.0001	0.8546	0.00004	18.324	0.001	38.532	0.004	15.66	0.001
25	UF-22692.666	Prigglitz-Gasteil, site "Cu I"	awl	2.0986	0.0001	0.85553	0.00004	18.28	0.002	38.362	0.011	15.639	0.002
27	UF-22692.1652	Prigglitz-Gasteil, site "Cu I"	clip (belt)	2.0958	0.0001	0.85107	0.00001	18.407	0.002	38.579	0.009	15.666	0.001
37	S001	Prigglitz-Gasteil, Klausgraben	axe (socketed)	2.0955	0.0001	0.85291	0.00001	18.343	0.002	38.438	0.016	15.645	0.002
38	S044	Bürg near Prigglitz-Gasteil, Klausgraben	axe (flat)	2.0764	0.0001	0.84152	0.00002	18.567	0.002	38.554	0.004	15.625	0.001
39	UF-22692.730	Prigglitz-Gasteil, site "Cu I"	copper ore*	1.7651	0.0001	0.69653	0.00001	22.785	0.001	40.218	0.002	15.87	0.001
42	UF-22692.2514	Prigglitz-Gasteil, site "Cu I"	copper ore*	1.8244	0.0001	0.72703	0.00002	21.756	0.001	39.693	0.005	15.817	0.001
45	UF-22692.2666	Prigglitz-Gasteil, site "Cu I"	copper ore*	1.6648	0.0001	0.66041	0.00002	24.132	0.001	40.174	0.005	15.937	0.001
48	UF-10796.2	Prigglitz-Gasteil, site "Cu I"	copper ore*	1.8465	0.0001	0.73096	0.00002	21.621	0.003	39.923	0.014	15.804	0.002
51	S002	Gloggnitz-Semmeringtunnelportal	axe (flat)*	2.0948	0.0001	0.85308	0.00001	18.335	0.001	38.408	0.002	15.641	0.001
58	S070	Grünbach-Gelände	axe (hammer-shaped)*	2.0772	0.0001	0.84302	0.00001	18.498	0.001	38.423	0.003	15.594	0.001
60	UF-19451	Grünbach-Gelände	casting cake	2.0868	0.0001	0.84621	0.00002	18.516	0.001	38.639	0.006	15.669	0.001
61	UF-19453	Grünbach-Gelände	axe (end-winged)	2.1081	0.0001	0.85831	0.00001	18.25	0.001	38.473	0.007	15.664	0.001
62	H001	Heufeld	casting cake	1.9854	0.0001	0.79054	0.00001	19.893	0.001	39.496	0.012	15.726	0.001
63	H003	Heufeld	casting cake	2.0169	0.0001	0.80947	0.00003	19.385	0.001	39.098	0.007	15.691	0.001
67	3720 (S067)	Neunkirchen-Klosteräcker	casting cake*	1.9895	0.0001	0.80454	0.00001	19.504	0.001	38.802	0.005	15.692	0.001
70	9029 (S065)	Neunkirchen-Klosteräcker	casting cake*	1.9496	0.0001	0.77639	0.00001	20.284	0.001	39.545	0.005	15.748	0.001
72	72.485	Pottschach	knife	2.0932	0.0001	0.85033	0.00001	18.414	0.001	38.544	0.007	15.658	0.001
73	72.487 A	Pottschach	pin (type Vasenkopf)	2.104	0.0001	0.85522	0.00001	18.31	0.001	38.524	0.01	15.659	0.001
76	72.489	Pottschach	bracelet*	1.9579	0.0001	0.77474	0.00001	20.349	0.001	39.843	0.004	15.765	0.001
83	UF-9958	Prein, site V	awl	2.1054	0.0001	0.85569	0.00003	18.297	0.001	38.522	0.002	15.657	0.001
84	UF-9723.1	Prein, site II	copper ore*	1.9148	0.0001	0.76369	0.00001	20.645	0.001	39.531	0.003	15.766	0.001
88	not inv. (Fn. 24, S004, S041)	Reichenau-Kammerwandgrotte	chisel	2.1044	0.0001	0.85537	0.00002	18.314	0.001	38.539	0.008	15.665	0.001
89	not inv. (S010)	Reichenau-Kammerwandgrotte	copper (fragment)	2.081	0.0001	0.84068	0.00001	18.645	0.001	38.801	0.007	15.675	0.001
98	UF-4959 (S012)	Sieding-Hügel 1	ring	2.104	0.0001	0.85542	0.00002	18.304	0.001	38.512	0.005	15.658	0.001
99	UF-5098	Sieding-Murrer	axe (socketed)	2.1015	0.0001	0.85319	0.00001	18.362	0.001	38.587	0.008	15.666	0.001
100	UF-6117 (S023)	Sieding-Steinparzkreuz	casting cake	2.0907	0.0001	0.83412	0.00001	18.797	0.002	39.299	0.017	15.679	0.002

The samples were measured at 180 ppb 50ul/min* and 100 ppb 50ul/min with Aridus.

## 4. Discussion

### 4.1 Chemical composition

Roughly half of the objects contain ~ 7–12% Sn, which is the ideal range for casting and later cold deformation [42]. Contrarily, the four Neolithic axes (nos. 38, 51, 58, 66) have < 0.2%. Three objects from Prigglitz (nos. 12, 16, 33) contain significantly more Sn at 15–16 wt.%, and, peculiarly, some have > 1 wt.% Zn. No specific functional or typological groupings were found within the object corpus, with only the pins from Prigglitz being somewhat similar apart from no. 36, which contains 1.6 wt.% Zn and 0.5 wt.% Sn. However, the chemical groupings in the Ni-Ag diagrams may indicate the use of similar types of copper ores or recycled material. Fig 4 shows the logarithmic plots of Ni and Ag trace elements from sources relevant to the Prigglitz site. Nickel and Ag were chosen since they are more stable during metallurgical processes such as recycling compared to As and Sb.

**Fig 5:**
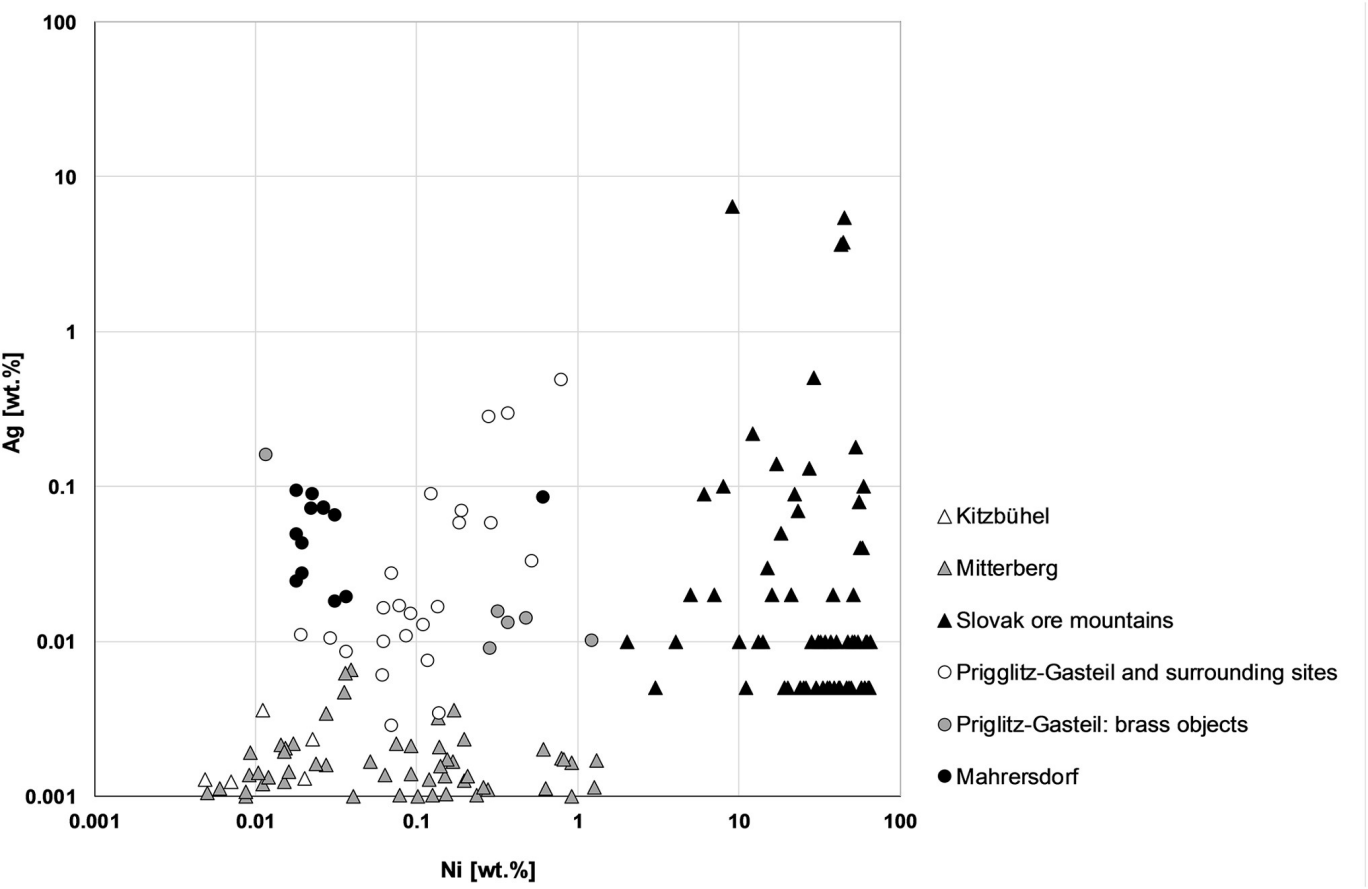
Logarithmic plots of Ni and Ag trace elements of copper ores from different sources. The values are normalized to copper and based on regional, interdisciplinary investigations of the specific mining regions [9, 36, 45, 46].

Data for the finds from the MBA site of Sieding plot together in the Ni-Ag diagram apart from ring no. 98 (Fig 5). Similar amounts of Ni and Ag are also characteristic of most of the Pottschach finds. The four casting cakes from Heufeld form a second distinct group below, and the remainder, especially those from Gasteil, are widely dispersed. Of note, the Grünbach-finds tend to contain relatively high amounts of Ni and Ag. All but three Grünbach finds, two copper fragments and the hammer-shaped axe, plot similarly. The objects from Gasteil that contain significant amounts of Zn, over 0.9 wt.%, are characterized by comparably low amounts of Ag and high Ni. The only exception is pin no. 36, which contains higher amounts of Ag and low Ni. Examining the casting cakes closer, four from Heufeld and two from Grünbach form distinctive groups with those from Heufeld that contain less Ag. Interestingly, the two cakes from Grünbach are chemically dissimilar to the copper fragments from the same findspot. Casting cake and copper fragments from Reichenau also show comparably low concentrations of Ag. The Neunkirchen casting cakes show similar amounts of Ag, but their Ni content varies. Finally, element plots of the casting cakes from Prigglitz-Gasteil show discrepancies in Ni-Ag composition compared to data from mining sites in Slovakia and Tyrol/Salzburg. These differences suggest that the finds from Southeastern Lower Austria derived from several mines; similar observations have already been made for the LBA objects deposited at Mahrersdorf near Prigglitz-Gasteil [36].

**Fig 6:**
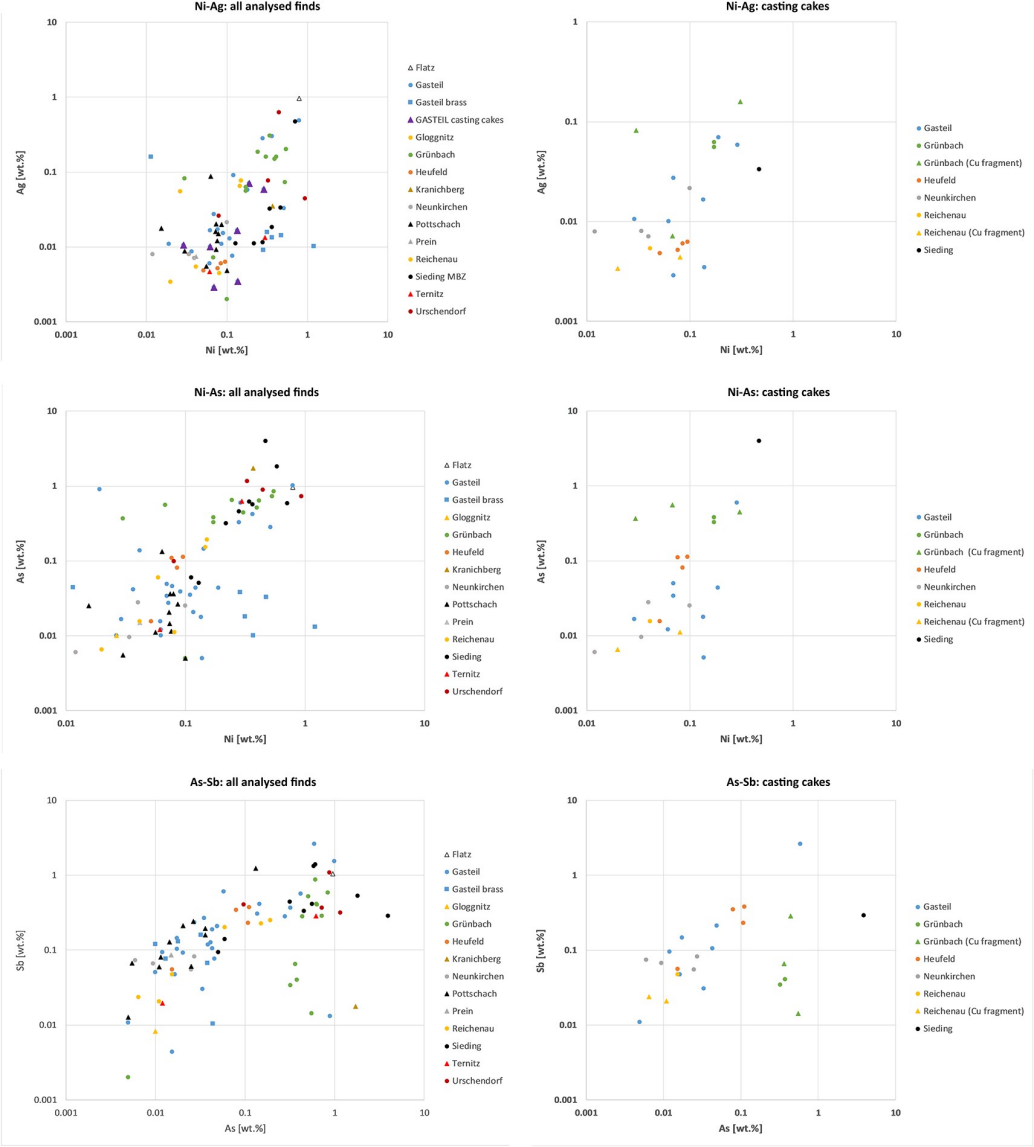
Logarithmic plots of Ni and Ag, Ni and As, and As and Sb trace elements.

The chemical data in the Ni-As diagram plots similarly to that of Ni-Ag shown in Fig 6. The MBA finds from Sieding plot close to each other with only the socketed axe showing less As. The Heufeld casting cakes (nos. 62, 63) also form a distinctive group, as do most of the Pottschach finds except bracelets nos. 76, 78, 81, and knife no. 72. All the objects from Grünbach show similar amounts of as with varying Ni, and the Prigglitz finds are widely dispersed in the diagram. The objects from Prigglitz-Gasteil that contain significant amounts of Zn, > 0.9 wt.%, form a distinct group with high amounts of Ni and comparably low As. The only exception to this pattern is pin no. 36, which contains high Ag and lower Ni concentrations. The casting cakes from Heufeld form a distinct group, with one showing noticeably less As. The cakes from Grünbach also have less As. However, the Ag and As in the casting cakes from Neunkirchen, Grünbach, and Prigglitz-Gasteil vary widely. The MBA casting cake from Sieding shows the highest amounts of both As and Ni.

The As-Sb diagram shows distinct groups for the Pottschach finds, excluding the bracelets (nos. 76, 78) and a knife (no. 72). The Grünbach finds have similar As but highly varying Sb, and the Heufeld finds, again, form a distinct group with similar As and Sb. Notably, the Grünbach hammer-shaped axe (no. 58) has significantly less As and Sb than the other Grünbach finds. As already noted in the other two logarithmic diagrams, the plots for the finds from Prigglitz-Gasteil are widely distributed and have highly variable As and Sb except for the objects that contain > 0.9 wt.% Zn, which, other than pin no. 36, form a distinct group. While the four Heufeld casting cakes show similar amounts of Ni and Ag, the As and Sb amounts of one of them (no. 65) is lower than the others. The casting cakes and the copper fragments from Grünbach have similar As, but differ widely in their Sb. The Neunkirchen casting cakes instead are more uniform in their As and Sb concentrations.

### 4.2 Brass production or mixing at Prigglitz-Gasteil

Five objects from Prigglitz contain 1–6.5 wt.% Zn, which is significantly higher than the ~ 0.1% measured in the local ores. These five objects include one casting drop with 0.9 wt.% (no. 10), a pin fragment with 1.4 (no. 36), a tubelet with 1.6 wt.% (no. 32), and two awls with 4.9 and 6.5 wt.% (nos. 24, 25). These objects demand closer consideration since metallic Zn production is complicated, requiring specialized furnaces and metallurgical knowledge. For instance, under extreme reducing conditions, Zn may be condensed to zinc oxide (ZnO); however, evidence for this method is unaccounted for in European prehistory. For percent concentrations of Zn to have been obtained, metalsmiths would have likely had to co-smelt Zn and Cu oxides. For instance, malachite co-smelted and leaded smithsonite can produce brass with ~ 15% Zn [47]. While direct evidence for co-smelting is lacking, its results are perhaps attested by a few hundred Zn-containing copper-based alloys (1–10% Zn) listed in the *Stuttgarter Metallanalysen Datenbank* (SAM) database [13].

Zinc oxide can also be extracted by roasting ZnS ores and Zn-containing fahlores. Zinc-sulfide ores and smithsonite have been found (Mindat.org [65]) ~ 60 km southwest of Prigglitz at medieval Pb and Zn mines in Arzberg, Styria [48]. A little further away, at ca 130 km from Prigglitz-Gasteil, sphalerite deposits occur in the Styrian district of Liezen (near Walchen and Trieben); in this region, LBA copper production is attested, and slag samples from LBA sites in Paltental Valley and Johnsbachtal Valley showed considerable trace amounts of Zn [49, 50]. Genetically similar Pb-Zn-Ag mineralizations also occur at Burgstaller Höhe and Haufenreith in the vicinity of Arzberg. Other Zn bearing (copper) ores, such as Zincolivenite (CuZn(AsO_4_)(OH), common in Brixlegg, Schwaz, and the Schwarzwald, or Zincrosasite ((Zn,Cu)_2_(CO_3_)(OH)_2_, common in the Greek mines of Laurion and the Rudabánya Mountains in North-eastern Hungary, are, however, unlikely to be related to the metal production at Prigglitz.

Given the limited evidence and availability of Zn, it is possible that Zn-containing objects from Prigglitz are associated with the smithsonite deposits in Styria, which were mistaken for copper ores and unintentionally included in the smelting process. However, a more appealing theory, due to the high variability in composition seen throughout the studied objects, is that the varying Zn concentrations resulted from metal recycling and materials mixing from extra-regional sources. Mixing would account for the object’s chemical variabilities and different concentrations of Zn.

### 4.3 Lead isotope analyses

As noted recently by [51] and numerous others (e.g. [52]), the most salient issues in archaeological Pb isotope provenance studies lie in their inconsistent data publication and presentation and the lack of a standard methodological approach. For instance, in the past, select and incomplete data were made available, along with isotope ratios devoid of ^204^Pb, such that the results were not reproducible and incomparable to other similar studies, and above all, lacked important geochemical characterizations. The strength of Pb isotope provenancing in archaeology, as is true in the geosciences and other Pb isotope-reliant fields, lies not in the apparent matching of like-like isotopic ratios but the baseline geological ages of referenced ores. However, since 2010 there has been a concerted effort to standardize Pb isotope provenance studies in archaeology following the conventions practiced in the geosciences. The most important aspect of adopting these conventions is the judicious reporting of all data and a complete set of ^204^Pb ratios [53]. These data allow for the reproducibility of results, the building of more extensive and inclusive isotope databases, and the identification of geochemical age relationships invaluable for regional and extra-regional source and object data comparisons.

**Fig 7:**
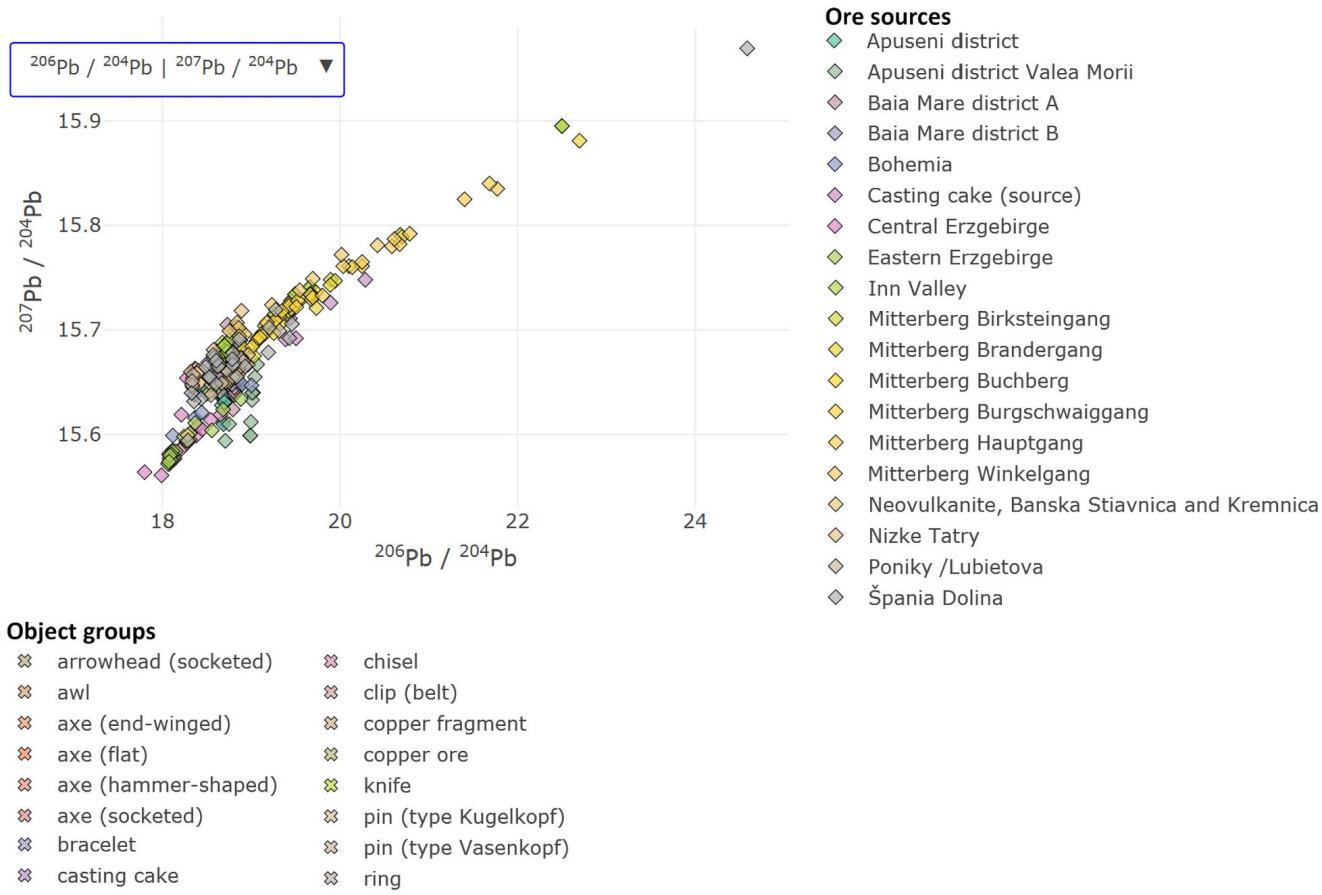
Bivariate lead isotope plot with isotope ratio comparison pulldown menu. The included supplemental data file allows for interactive zooming and data comparison, hiding data points, and saving still images. The data for these diagrams derive from [9, 44, 46, 54–58, 62, 63].

**Fig 8:**
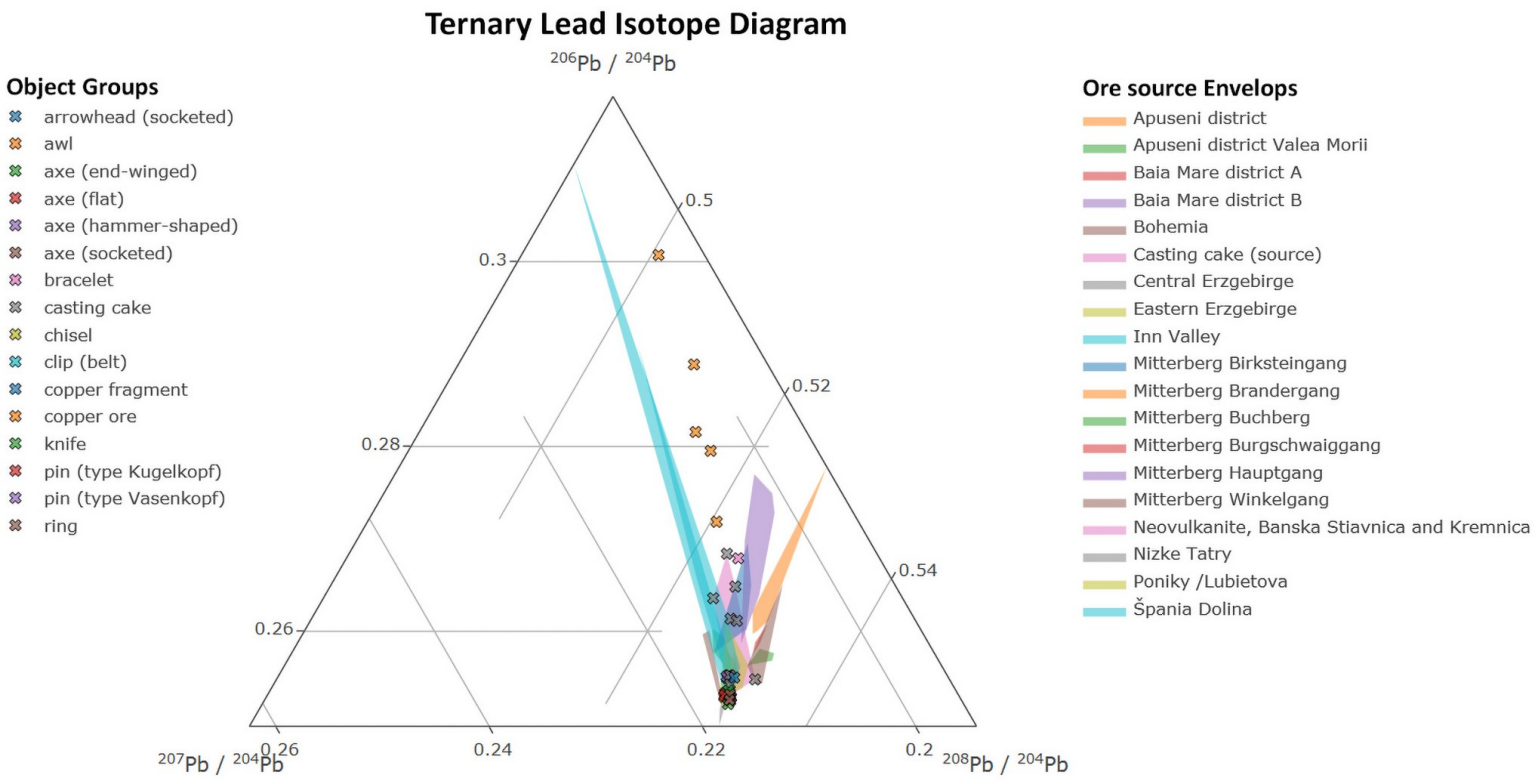
Ternary lead isotope plot with ore source envelopes. The included supplemental data file allows for interactive zooming and data comparison, hiding envelopes and data points, and saving still images.

In support of the standardization efforts mentioned above, a complete set of ^204^Pb-based isotopic ratios were calculated for Mitterberg and Slovakia from published data [9, 46] and are included in Table 3 and the accompanying online supplemental data files. These data, along with those published by [54–58], were used for comparison to those obtained in this work. The combined isotope data were plotted in interactive bivariate and ternary diagrams (Figs 7 and 8), using ^204^Pb-based ratios, to enhance the data’s interpretation and help identify mixing and source-object relationships. The diagrams were created in R using the plotly library and its dependencies [59, 60]. The ternary diagram is the result of the summation of the ratios equated to 1 [61], which are plotted in barycentric space and listed with the original isotope values in the interactive hover text. These diagrams are included in the online supplementary materials and open with any modern web browser.

Based on the interactive plots, materials used to produce the objects in this study may have originated from one or a combination of several possible ore sources. Furthermore, objects that appear between or next to the plotted ore envelopes, as depicted in Fig 7, may belong to adjacently positioned ore sites, but due to ore isotopic randomness across orebodies, are presented as incomplete overviews of the ore isotopic data range. For objects that lie within ore envelopes, several possible ores and materials could be considered as potential sources. Table 4 lists the observed direct source-object matches, which often overlap several ore envelopes, as shown in the ternary diagram.

**Table 4:** Observed object-site matches, which overlap within several ore envelopes in the ternary diagram.

No.	Object(s)	find spot	Source material										
			Apuseni district	Baia Mare district A	Casting cake (source)	Central Erzgebirge	Eastern Erzgebirge	Mitterberg Birksteingang	Mitterberg Winkelgang	Špania Dolina	Neovulkanite	Nizke Tatry	Poniky/Lubietova
3	Casting cake	Prigglitz-Gasteil					x					x	
5	Casting cake	Prigglitz-Gasteil, site "Cu I"	x				x			x	x		x
12	Arrowhead	Prigglitz-Gasteil, site "Cu I"	x		x	x	x			x	x		x
13	Arrowhead	Prigglitz-Gasteil, site "Cu I"					x					x	
15	Ring	Prigglitz-Gasteil, site "Cu I"			x		x			x	x	x	
18	Pin (Kugelkopf)	Prigglitz-Gasteil, site "Cu I"				x	x						
23	Awl	Prigglitz-Gasteil, site "Cu I"					x					x	
25	Awl	Prigglitz-Gasteil, site "Cu I"				x	x						
27	Clip	Prigglitz-Gasteil, site "Cu I"			x		x			x		x	x
37	Axe (socketed)	Prigglitz-Gasteil, Klausgraben				x	x			x			
38	Axe (flat)	Bürg near Prigglitz-Gasteil, Klausgraben	x		x	x	x			x	x		x
51	Axe (flat)	Gloggnitz				x	x			x			
58	Axe (hammer-shaped)	Grünbach			x	x	x			x			x
60	Casting cake	Grünbach					x			x	x		x
62	Casting cake	Heufeld						x					
63	Casting cake	Heufeld						x					
67	Casting cake	Neunkirchen								x			
72	Knife	Pottschach					x			x			x
89	Copper fragment	Reichenau-Kammerwandgrotte	x	x	x					x	x	x	x
99	Axe (socketed)	Sieding-Murrer										x	
100	Casting cake	Sieding							x				

The object isotopic signature plots, along with those immediately adjacent to envelopes or between them, may, in fact, belong to multiple sources, which is most readily explained by source and metal material mixing or possible related geochemical characteristics among the considered ores. However, given the chemical variability of the objects discussed in this paper, it is likely that the mixing of new and recycled metal resulted in the aggregation of similar Pb isotope ratios towards the bottom of the ternary plot where the Pb was most abundant and the isotopes most concentrated. Of note, the copper ores from Prigglitz-Gasteil and Prein an der Rax plot well outside the ore envelopes and contain very little Pb. Their mixing with any higher Pb-containing materials, such as other ores, scrap metal, or refined Pb, would have resulted in the copper ores’ original isotopic signatures being overwritten. As a result, any subsequently created objects would have been isotopically dissimilar to the added source material(s).

The ternary diagram, and less obviously, the bivariate, show several possible ore source possibilities; however, it is important to note that few Pb isotope measurements of regional copper minerals have been undertaken, which could later impact the studied objects’ interpretation to the available source materials. This information may also illuminate the local and regional ores’ relationship and whether they are ultimately helpful in discriminating source materials or if they share a common isotopic range. Notwithstanding this missing information, what is clear from the diagrams is that mixing occurred. In addition to the copper ore isotopic signatures mentioned above, the differences, or more likely, shared geochemical character of the Eastern and Central Erzgebirge, may be isotopically indistinguishable. As shown in Fig 7, the envelopes for these sources overlap and contain 7 of the studied objects (nos. 12, 18, 25, 37, 38, 51, 58), with the majority of the corpus lying on or just outside the boundaries of the more expansive range of the Eastern Erzgebirge. These similarities, along with the overlapping and encroaching Nizke Tatry and Neovulkanite, Banska Stiavnica, and Kremnica [46] envelopes, further suggest orebody age similarities and the necessity for additional study of the region’s geology and archaeological materials.

A more pronounced example of the above is arrowhead no. 12, which lies within several source material envelopes, including; the Eastern and Central Erzgebirge, Apuseni District, Špania Dolina, and Poniky / Lubietova. The arrowhead also lies within the pseudo casting cake ore source envelope, suggesting the object’s material life history may have included an iterative casting cake precursor. The casting cakes, plotted in Fig 7 as both objects and a pseudo ore source, may have resulted from the mixing of ores, metals, and other Pb-containing materials that extended beyond the enclosed space shown in the diagram. Notably, the casting cakes, treated individually, plot within several ore source envelopes, giving credence to the belief that many are the product of mixing. In fact, only casting cake no. 70 may have come from a single orebody, while the others appear to have originated from a combination of sources. Given these data, future analyses must include additional casting cakes to determine their relation, as first iteration objects or the result of combined source materials, before the life histories of the objects made thereof can be understood.

## 5. Conclusions

### 5.1 Characterization of the samples from Prigglitz-Gasteil

According to previous mineralogical investigations by Michael Götzinger and Uwe Kolitsch (pers. commun.; see also [19, 64], the **ore deposit** at the Prigglitz-Gasteil site (known as "Sandriegel" in the mineralogical literature) is dominated by chalcopyrite, pyrite, and siderite with minor occurrences of fahlore and secondary minerals (malachite, azurite, and limonite). One of the ore’s most striking characteristics, and 4 others from Prein an der Rax (chalcopyrite, fahlore, malachite, and azurite), are their almost complete lack of Pb (averaging 5 ppm Pb with a maximum of 12.1). As for the objects analysed in this paper, with the exception of casting cake no. 70, it seems that they were subject to some form of mixing, which must be the case since they contain measurable amounts of Pb.

Since many of the copper ores in this study do not contain appreciable amounts of Pb, any copper-based objects with measurable Pb isotopic ratios must have resulted from the mixing of source materials that originated outside of Prigglitz-Gasteil, or the objects were primarily the product of other sourced ores. Fig 7 shows that those ores with measurable isotopes lie far outside the Prigglitz ore source envelopes, suggesting that, even if local copper ores were exploited, extra-regional mixing metal-containing-lead was prevalent at the site. Indeed, four out of seven casting cakes from Prigglitz-Gasteil contain no detectable Pb and are otherwise composed of considerably pure copper (Ni max. 0.14%, As max. 0.034%, Ag max. 0.017%, and Sb max. 0.047%) with 0.95–5.81 wt.% Fe. They were most likely smelted from chalcopyrite and almost certainly a product of Prigglitz-Gasteil ores.

For our project, only two of the casting cakes (nos. 3 and 5) from Prigglitz-Gasteil with lead > 0.012% were analysed for their Pb isotopic ratios due to the element’s scarcity. In the future, when more sensitive analytical equipment becomes available, those that remain should be reinterpreted. Thus, with the data at hand, the two main characteristics of the Prigglitz-Gasteil chalcopyrite copper, its purity and lack of Pb, prevent a straightforward attempt to identify or eliminate their associated source materials. Furthermore, the measured trace elements, which may be present due to the mixing of ores, in addition to the complex chemical character of the ores individually, make tracing the Prigglitz-Gasteil copper untenable at this time. The situation is especially complicated considering the possible local smelting of fahlores, as evidenced by ore sample no. 42 and casting cake no. 3.

Our chemical analyses also showed that several objects near Prigglitz-Gasteil contain Zn, which is tempting to interpret as indicative of brass production in the immediate vicinity. However, based on its sporadic presence and variable concentrations, it is far more likely the result of the mixing of higher content Zn alloys of uncertain origin with locally produced copper. Therefore, the Zn-containing objects at the site are more likely indicative of an active metal recycling effort near or at Prigglitz-Gasteil, and the mixing of old metal with newly produced Cu, rather than a purposeful effort to alloy Cu with Zn.

### 5.2 Identifying copper products originating from the Prigglitz-Gasteil mine

Despite the interpretive difficulties mentioned above, trace element similarities between the casting cakes and ores hint that some objects were produced from Prigglitz copper. Among the LBA copper objects found at six sites within a radius of c. 15 km around Prigglitz-Gasteil, one casting cake and two casting residues from Kammerwandgrotte Cave (nos. 89, 90, 92) and the casting cakes from Neunkirchen (except no. 68) align with the Prigglitz’s mine ore chemistry. However, while the trace element compositions are a convincing match, the Pb isotopic signatures neither overlap with the ores nor the casting cakes. Despite these incongruencies, it must be reiterated that only the objects with > 0.005% Pb, the detection limit of our XRF instrument, were selected for isotopic analysis. Thus, the presented Pb isotope data do not fully represent the scope of the Prigglitz-Gasteil copper objects. Lead isotopic analyses of the low Pb-content objects, when conducted, may, in fact, align with the ores at Prigglitz-Gasteil. There are also similarly composed ores, with very little Pb, from the Prein an der Rax region, which should be accounted for in future analyses and interpretations.

Regarding the reconstruction of regional distribution networks of metal and materials, it is also useful to consider which copper objects did not originate from Prigglitz-Gasteil ores. First, the small casting cakes found in the Heufeld hoard near Gloggnitz almost certainly derive from a different copper source, as they contain more As (0.08), Sb (0.25), and Pb (0.006). The casting cake from the Mahrersdorf hoard must have also been produced from at least two different sources, along with the other copper-based objects found there [36]. One small casting cake and the copper-alloy jewelry from the MBA graves at Sieding have fahlore-like chemistry, which clearly differs from the Prigglitz-Gasteil ores. Finally, the chemistry of the finds at the Gelände hillfort near Grünbach am Schneeberg, where several hoards, casting cakes, and slags were found, indicates that the metal most likely originated from the Slovakian Ore Mountains.

The other sites investigated in the region surrounding Prigglitz-Gasteil did not yield copper casting cakes, so a reasonable comparison with the studied mining sites is problematic. Among the products investigated, trace element patterns from the nearby cemetery of Pottschach overlap with the Prigglitz-Gasteil objects, indicating a possible common raw material origin.

Regarding the distribution of copper and copper alloy objects produced at Prigglitz-Gasteil, a comparison of the chemical analyses indicated that they were likely exported to the Kammerwandgrotte Cave at Reichenau, the LBA lowland settlement at Neunkirchen, and the LBA community interred at the Pottschach cemetery. These results, and those discussed above, suggest that Prigglitz-Gasteil sourced metal or raw materials were exchanged in the Schwarza Valley’s micro-region at least as far as c. 11.5 km away. Additional investigations of more distant LBA sites will be necessary to fully explore the extent of exchange and the Prigglitz-Gasteil mining site’s role.

### 5.3 Identification of bronze objects of different origin at Prigglitz-Gasteil

All of the local ore samples from Prigglitz-Gasteil contain 12 ppm or less Pb; its presence in higher concentrations in the objects, therefore, likely indicates the inclusion of externally sourced ores or metal mixing and recycling. Twenty-one out of the 27 objects from Prigglitz-Gasteil contain more than 0.008% Pb, which is 1000 times more than the local ores. As already mentioned above, the Pb isotope signatures of the Prigglitz-Gasteil objects do not match the local ores, whose isotopic ratios would have changed dramatically with even small amounts of added Pb. Eight of the objects from Prigglitz-Gasteil reside within or near a set of envelopes for the Eastern or Central Erzgebirge (Slovakian Ore Mountains), Špania Dolina, and the Apuseni district in Romania. The most practical interpretation of these data is that the source materials originated from the closest source, the Slovakian Ore Mountains, situated c. 350 km from Prigglitz-Gasteil. The other sources are much more distant, with the Apuseni district being c. 700 km away. In general, fahlore copper from the Slovakian Ore Mountains is well attested during the LBA in southeastern Lower Austria, as indicated by the prevailing Pb isotope patterns from samples taken at Pottschach and Grünbach-Gelände.

One of the more interesting aspects of our analyses was the five objects from Prigglitz-Gasteil that contain Zn. As shown by the Ni-Ag logarithmic diagrams, four of the five Prigglitz-finds contain Zn and plot similarly. These data suggest that materials used to create these objects originated from a similar, if not the same, copper ore source. A possible source for zinc and these Zn-containing objects is Arzberg, Styria, located c. 100 km to the southwest of Prigglitz; however, there is currently no evidence for the exchange of raw materials or objects between these sites. It is also possible that the Zn content, being in lower concentrations than what is typically deemed a brass, is present due to the recycling and mixing of locally produced Cu with higher concentrated Zn-containing scrap alloys, as mentioned in section 5.1. These scrap alloys, originating elsewhere, would account for the variable Zn-content and overall altered chemistry of the objects while also significantly changing the Pb isotopic character of the objects at and surrounding Prigglitz-Gasteil.

### 5.4 The chaîne opératoire of copper production at Prigglitz-Gasteil

In the areas excavated from 2010–2014, the earliest product of the local *chaîne opératoire* of metal production is represented by several casting cakes of pure copper. No finds resulting from copper ore smelting (coarse slags) or intermediary products of the smelting process (matte or black copper) were found on terraces T3 or T4. Our analyses indicate that the objects produced with locally available chalcopyrite were likely mixed with materials from external origins to produce casting cakes and/or scrap metal during casting. It remains unclear whether pure tin (Sn), or Sn ores, were added to the local copper to produce bronze or if the presence of Sn resulted from the recycling and mixing of scrap metal. For Zn-containing objects, it is possible its presence was due to the mixing of fahlore-derived scrap metal, and in some instances, Zn seems to substitute for Sn, suggesting metal type recycling intentionality. In the Late Urnfield Period (mid-11^th^ to 9^th^ century BC), when most copper mines in the Eastern Alps and the Slovakian Ore mountains produced copper with fahlore, and almost all copper-based alloys in circulation were highly impure (i.e., with high percentages of Sb and As), the fresh, pure chalcopyrite-based copper from Prigglitz-Gasteil may have been recognized as important in controlling the concentration of alloying elements. Pure copper would have allowed for predictability in colour and physical property outcomes of intentionally made alloys. The 24 objects from Prigglitz-Gasteil perhaps illustrate the purposeful recycling of old metal. They are small tools, lost dress items, or fragments of other small objects that were, for the most part, concentrated in a small area of terrace T3, belonging to late occupation phases: T3-10 c. 905 BC; T3-08F c. 905–860 BC; and T3-08A c. 845–810 BC. They were found with numerous casting drops and fragments of stone casting molds used to produce knives with tang hilts *(Griffdornmesser)*, socketed axes, and weapons (socketed arrowheads); none of these finished objects were however found at the site. In other words, our analyses do not fully represent the metal character nor the intended final products produced at Prigglitz.

The archaeological context suggests that most of the small metal finds -fragmentary and broken- are collected scrap metal intended for recycling. The heterogeneity of these objects and that of the casting cakes, in terms of chemical composition and Pb isotopic ratios, supports this interpretation, as both fresh copper and scrap metal are present. As is true in modern bronze foundries, it is useful to have scrap metal on hand to mix with new metal to achieve desired alloying outcomes or when new metal supplies are insufficient. The process of heating and pouring molten metal is, after all, the same, whether done with new, old, or a mixture of metals. Furthermore, scrap recycling reduces the time and resources needed to produce the same objects while avoiding the most fuel-intensive step of primary smelting. Unfortunately, there is currently no way to estimate the proportion of locally produced or imported copper or imported final products at Prigglitz. However, the assemblage of copper alloy objects studied in this paper strongly suggests that localized recycling took place at the site in three phases (T3-10, T3-08F, T3-08A). These activities belong to late phases of metal production and operations of the Prigglitz-Gasteil mine, and almost certainly align with the site’s gradual decline during the late 10^th^/9^th^ centuries BC.

Given the data presented in this paper, the most practical conclusion is that Prigglitz-Gasteil was an active copper mining and metal-making site that also actively imported and recycled alloys. Since Prigglitz-Gasteil copper contains almost no detectable Pb, any measurable concentrations of the element are due to the inclusion of externally sourced materials, dramatically altering the objects’ isotopic ratios. According to the Pb isotopic ratios and proximity to the site, most of the recycled copper likely originated from the Slovakian Ore Mountains. We also note that many object isotopic signatures do not reside in any of the plotted envelopes, suggesting insufficient ore sampling or contributions from additional ore sources such as the Gschriebenstein near the LBA metal production centre Velem-Szentvid in Western Hungary. There is, therefore, the need to characterize more copper ore deposits in the surroundings of the Vienna Basin, especially those in western Hungary, northern Styria, and northwest Slovenia. Another necessary step in future investigations will be to analyse the low Pb objects and ores for comparison to those from Prigglitz-Gasteil and surrounding sites. With this data, it may then be possible to make more definitive conclusions on the role of Prigglitz-Gasteil on metal production in the region.

## Supporting information

S1 Data (DOCX)

S1 Plot (HTML)

S2 Plot (HTML)
