# Supplementary material for: Melting, smelting, and recycling: A regional study around the Late Bronze Age mining site of Prigglitz-Gasteil, Lower Austria
Source: PLoS One. 2021 Jul 16;16(7):e0254096. doi: 10.1371/journal.pone.0254096 (PMC8284822; doi:10.1371/journal.pone.0254096)
Supplement: S1 Data — (DOCX) [file pone.0254096.s001.docx]

**S1 Data**

**S1 and S2 are both html files.**

**S1: Bivariate lead isotope plot with isotope ratio comparison pulldown menu.** The included supplemental data file allows for interactive zooming and data comparison, hiding data points, and saving still images. The data for these diagrams derive from: Baumann et al. (2000); Breiter (1982); Hyrsl (1992); Jarchovsky et al. (1994); Marcoux et al. (2002); Niederschlag et al. (2003); Pernicka et al. (2016); Schreiner (2007); Schubert (2005); and Žák et al. (1991).

**S2: Ternary lead isotope plot with ore source envelopes.** The included supplemental data file allows for interactive zooming and data comparison, hiding envelopes and data points, and saving still images. The data for these diagrams derive from: Baumann et al. (2000); Breiter (1982); Hyrsl (1992); Jarchovsky et al. (1994); Marcoux et al. (2002); Niederschlag et al. (2003); Pernicka et al. (2016); Schreiner (2007); Schubert (2005); and Žák et al. (1991).
